# Comprehensive mapping of the AOP-Wiki database: identifying biological and disease gaps

**DOI:** 10.3389/ftox.2024.1285768

**Published:** 2024-03-08

**Authors:** Thomas Jaylet, Thibaut Coustillet, Nicola M. Smith, Barbara Viviani, Birgitte Lindeman, Lucia Vergauwen, Oddvar Myhre, Nurettin Yarar, Johanna M. Gostner, Pablo Monfort-Lanzas, Florence Jornod, Henrik Holbech, Xavier Coumoul, Dimosthenis A. Sarigiannis, Philipp Antczak, Anna Bal-Price, Ellen Fritsche, Eliska Kuchovska, Antonios K. Stratidakis, Robert Barouki, Min Ji Kim, Olivier Taboureau, Marcin W. Wojewodzic, Dries Knapen, Karine Audouze

**Affiliations:** ^1^ Université Paris Cité, Inserm UMR-S 1124 T3S, Paris, France; ^2^ Norwegian Institute of Public Health, Division of Climate and Environment, Oslo, Norway; ^3^ Department of Pharmacological and Biomolecular Sciences, Università degli Studi di Milano, Milan, Italy; ^4^ Zebrafishlab, Department of Veterinary Sciences, Veterinary Physiology and Biochemistry, University of Antwerp, Wilrijk, Belgium; ^5^ Institute of Medical Biochemistry, Medical University of Innsbruck, Innsbruck, Austria; ^6^ Institute of Bioinformatics, Biocenter, Medical University of Innsbruck, Innsbruck, Austria; ^7^ Department of Biology, University of Southern Denmark, Odense, Denmark; ^8^ Environmental Engineering Laboratory, Department of Chemical Engineering, Aristotle University of Thessaloniki, Thessaloniki, Greece; ^9^ National Hellenic Research Foundation, Athens, Greece; ^10^ Science, Technology and Society Department, Environmental Health Engineering, University School for Advanced Studies (IUSS), Pavia, Italy; ^11^ Department II of Internal Medicine, Faculty of Medicine and University Hospital Cologne, University of Cologne, Cologne, Germany; ^12^ Center for Molecular Medicine Cologne, Faculty of Medicine and University Hospital Cologne, University of Cologne, Cologne, Germany; ^13^ Cologne Excellence Cluster on Cellular Stress Responses in Aging-Associated Diseases (CECAD), Cologne, Germany; ^14^ European Commission, Joint Research Centre (JRC), Ispra, Italy; ^15^ IUF-Leibniz Research Institute for Environmental Medicine, Duesseldorf, Germany; ^16^ Heinrich-Heine-University, Düsseldorf, Germany; ^17^ Swiss Centre for Applied Human Toxicology, Basel, Switzerland; ^18^ DNTOX GmbH, Düsseldorf, Germany; ^19^ Inserm UMR-S 1124, Université Sorbonne Paris Nord, Bobigny, Paris, France; ^20^ Université Paris Cité, BFA, Team CMPLI, Inserm U1133, CNRS UMR 8251, Paris, France; ^21^ Cancer Registry of Norway, NIPH, Oslo, Norway

**Keywords:** adverse outcome pathway, AOP network, immunotoxicity, neurotoxicity, nongenotoxic carcinogenesis, diseases, PARC

## Abstract

**Introduction:** The Adverse Outcome Pathway (AOP) concept facilitates rapid hazard assessment for human health risks. AOPs are constantly evolving, their number is growing, and they are referenced in the AOP-Wiki database, which is supported by the OECD. Here, we present a study that aims at identifying well-defined biological areas, as well as gaps within the AOP-Wiki for future research needs. It does not intend to provide a systematic and comprehensive summary of the available literature on AOPs but summarizes and maps biological knowledge and diseases represented by the already developed AOPs (with OECD endorsed status or under validation).

**Methods:** Knowledge from the AOP-Wiki database were extracted and prepared for analysis using a multi-step procedure. An automatic mapping of the existing information on AOPs (i.e., genes/proteins and diseases) was performed using bioinformatics tools (i.e., overrepresentation analysis using Gene Ontology and DisGeNET), allowing both the classification of AOPs and the development of AOP networks (AOPN).

**Results:** AOPs related to diseases of the genitourinary system, neoplasms and developmental anomalies are the most frequently investigated on the AOP-Wiki. An evaluation of the three priority cases (i.e., immunotoxicity and non-genotoxic carcinogenesis, endocrine and metabolic disruption, and developmental and adult neurotoxicity) of the EU-funded PARC project (Partnership for the Risk Assessment of Chemicals) are presented. These were used to highlight under- and over-represented adverse outcomes and to identify and prioritize gaps for further research.

**Discussion:** These results contribute to a more comprehensive understanding of the adverse effects associated with the molecular events in AOPs, and aid in refining risk assessment for stressors and mitigation strategies. Moreover, the FAIRness (i.e., data which meets principles of findability, accessibility, interoperability, and reusability (FAIR)) of the AOPs appears to be an important consideration for further development.

## 1 Introduction

In 2007, the US National Research Council (NRC) published a review ‘Toxicity testing in the 21st century: a vision and a strategy’, highlighting the ongoing paradigm shift in toxicity testing (Krewski et al., 2020). Indeed, existing risk assessment methodologies and toxicological tests are not in line with the large number of not sufficiently tested existing and the rapidly growing number of novel substances, including metabolites produced in the environment by non-humans, that need to be evaluated. Consequently, the potential toxicity of many substances is poorly characterized. Moreover, the diversity of the organ systems being targeted by contaminants or pollutants, increases the need to implement new tests. Therefore, in this study, authors suggested recommendations to improve and accelerate chemical testing, and emphasized the concept of ‘toxicity pathways’ (Ankley et al., 2010). The development and use of New Approach Methodologies (NAMs), which include animal and non-animal-based methods, have increased to assess specific endpoints that indicate human and ecotoxicological risks from exposure to environmental substances. NAMs are developed to identify a toxicologically relevant response at different biological levels (molecular, cellular, organ, organism) provoked by a chemical exposure (Vinken et al., 2017). NAMs include *in cellulo, in vitro* and *in chemico* screening, computational methods as well as high throughput multi-omics and exposomics (Babin et al., 2023; Maitre et al., 2023) to mention but a few methodologies. Recently, a study explored the progress in this ‘next-generation’ of risk assessment methodologies, and highlighted a low, but still growing evolution and use of NAMs in toxicity testing and risk assessment (Krewski et al., 2020). Moreover, limitations of the use of animals in regulatory studies are also an important component to take into consideration, as animal OECD test guideline studies are time- and cost-intensive. Furthermore, it is increasingly ethically questionable (testing one substance may require several thousand animals) in addition to the uncertainties in methodologies, evaluation, regulation, and extrapolation. Although still assumed to be the most protective types of study, *in vivo* animal study predictivity for the protection of human health is sometimes insufficient given the differences in organ function/complexity, exposure, developmental timing, toxicokinetics and toxicodynamics between humans and other mammals (Tsuji and Crofton, 2012). In the recent assessment of Extended One-Generation Reproductive Toxicity Studies (EOGRTS; OECD TG 443) conducted with 55 industrial chemical substances under REACH (European Chemicals Agency, 2023), methodological issues were presented concerning the developmental immuno- and neurotoxicity cohorts that appear to be particularly demanding in terms of proficiency, i.e., deficiencies were frequently found that hindered the interpretation of the results. This, again, highlights the need for methods’ development to support risk assessment of chemicals by considering human biology and providing a deep mechanistic understanding of the toxicological events leading to adverse effects.

To facilitate the use of NAMs in a regulatory context, a new concept was proposed by Ankley in 2010, named Adverse Outcome Pathways (AOP) (Ankley et al., 2010). The AOP framework describes and organizes existing biological knowledge for humans and wildlife from the perturbation by a stressor leading to an adverse effect, in a structured manner. An AOP starts with a Molecular Initiating Event (MIE) (initiated by a prototypical stressor, the latter not being part of the AOP), linked to a sequence of biological Key Events (KE) that ultimately lead to an Adverse Outcome (AO) at the level of an individual, population or ecosystem. One main objective is to support chemical risk assessment based on mechanistic reasoning and regulatory toxicology. In 2013, the Organization for Economic Co-operation and Development (OECD) started an initiative for the formalization of AOP development, resulting in guidance documents (OECD, 2017; OECD, 2018). Today, AOPs are becoming more and more acceptable as a framework to help chemical safety assessment and regulatory toxicology by supporting a systematic way of predicting AOs based on accumulated mechanistic knowledge (Svingen et al., 2021). To follow the regulatory framework proposed by the European Union (EU) to reduce levels of environmental stressors (e.g., Endocrine Disrupting Chemicals (EDC)), research-driven approaches need to be developed, which include AOP-informed Integrated Approaches for Testing and Assessment (IATA) as an imperative feature of the risk assessment process. The European Cluster to Improve Identification of Endocrine Disruptors (EURION, https://eurion-cluster.eu) (Street et al., 2021) as initiated a few years ago with the aim to propose new and effective methods for EDC testing, related to several health outcomes such as metabolic disorders (Audouze et al., 2020) or developmental neurotoxicity (DNT) (Lupu et al., 2020). Likewise, the ASPIS cluster (https://aspis-cluster.eu/) uses AOP-based approaches for implementation of novel strategies for animal-free safety assessment of (non-endocrine) chemicals. Furthermore, the Partnership for the Assessment of Risks from Chemicals initiative (PARC) has the ambition to develop a robust risk assessment of new generation chemicals to better protect health and the environment (https://www.eu-parc.eu/) (Marx-Stoelting et al., 2023). The development of AOPs is a key feature of PARC. Previously, several studies have described both the development of AOPs and their potential uses for risk assessment (Villeneuve et al., 2014; Leist et al., 2017; Villeneuve et al., 2018a; Knapen et al., 2018; Vinken, 2018; Bajard et al., 2023). Evidence-based approaches (Audouze et al., 2021) and innovative tools have been proposed to help in the construction of AOPs such as the AOP-helpFinder tool that is based on artificial intelligence (http://aop-helpfinder.u-paris-sciences.fr/) (Carvaillo et al., 2019; Jornod et al., 2022; Jaylet et al., 2023). AOP-helpFinder automatically screens the available literature and provides a very important source of knowledge that can be used for AOP development (Benoit et al., 2022; Jaylet et al., 2022). Since 2014, all developed AOPs are stored in the AOP-Wiki database (https://aopwiki.org/) that is part of the AOP-knowledge-base (AOP-KB; https://aopkb.oecd.org) set up by the OECD. For each proposed AOP, KEs should be measurable, meaning that associated test methods should need to be mentioned if they exist, or developed if they do not. Therefore, the development of AOPs follows the current availability of test methods. For example, a recent study focusing on EDCs, highlighted that validated *in vitro* and *in vivo* tests used by the EU to identify the potential toxicity of EDCs do not assess all the endocrine pathways (Zgheib et al., 2021). From these findings, an online tool has been developed to evaluate the status of their tests under development before submission to the OECD for endorsement (https://readedtest.u-paris-sciences.fr/).

The current study aims in a holistic approach across the whole content of the AOP-Wiki to identify gaps in the current AOPs within the AOP-Wiki database for future research needs. It does not intend to provide a systematic and comprehensive summary of the available literature, but rather summarizes and maps the biological knowledge and diseases represented by the already developed AOPs (OECD endorsed status or still under validation).

## 2 Material and methods

**FIGURE 1:**
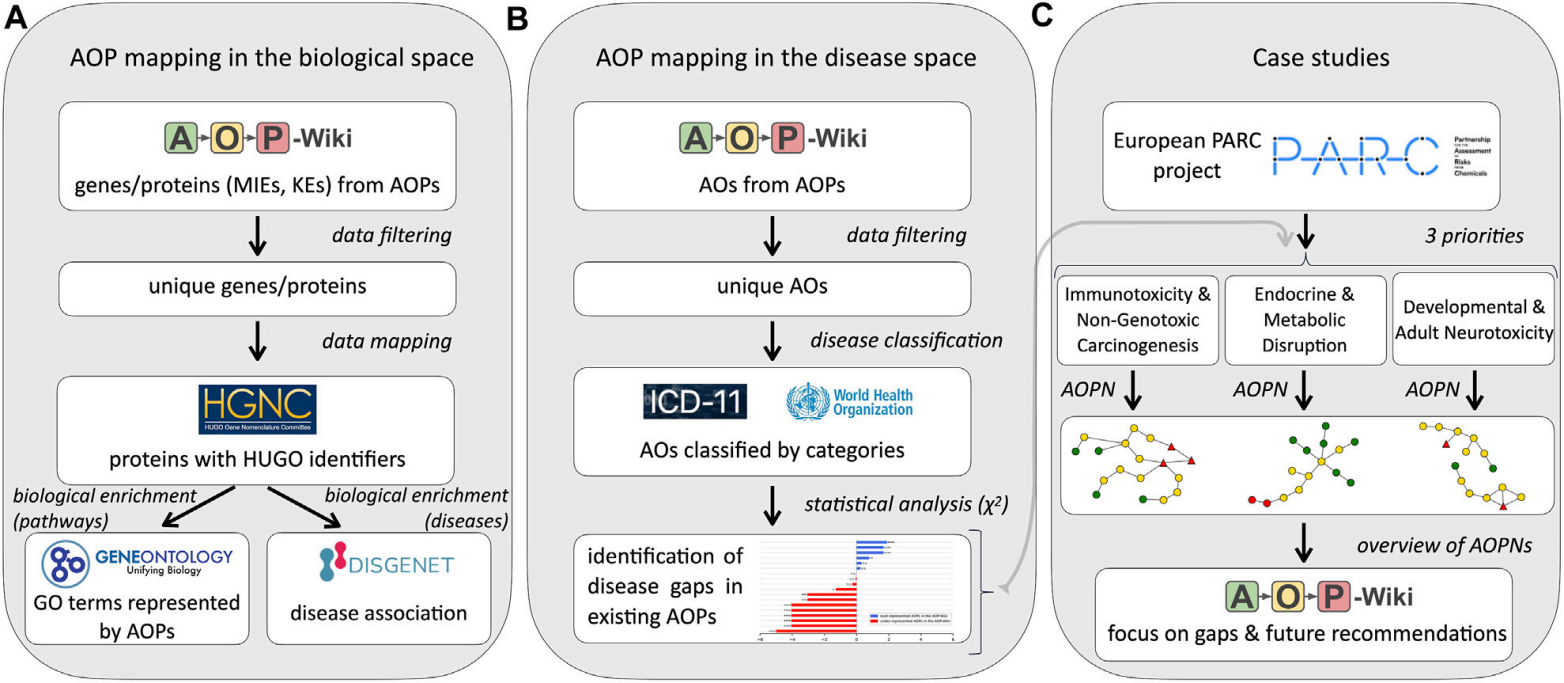
Workflow of the procedure developed to assess the global mapping of existing Adverse Outcome Pathways (AOPs). For each evaluation, a comprehensive workflow was developed: **(A)** Workflow for AOP and the biological space, **(B)** Workflow for AOP and disease space and **(C)** Workflow for the three case studies. MIE: Molecular Initiating Event, KE: Key Event, AO: Adverse Outcome, AOPN: Adverse Outcome Pathway Network, HGNC–HUGO gene Nomenclature Committee, ICD-11–International Classification of Diseases 11th Revision, GO: Gene Ontology.

The proposed approach, to explore information related to AOPs from the AOP-Wiki (i.e., at the gene/protein levels and the disease level), is a multi-step procedure illustrated in Figure 1.

### 2.1 Compilation and preparation of AOP information from the AOP-Wiki database

For both mapping approaches (genes/proteins and adverse outcomes), the AOP-Wiki database was used. The AOP-Wiki compiles data of all existing AOPs submitted by researchers and are either under development or have been endorsed by the OECD. By the time of our analysis, it contained 403 unique AOPs, but continues to increase rapidly due to global efforts based on crowdsourced collaboration. However, until now only 29 AOPs have been endorsed by the OECD, the others being under development or under evaluation. In this study, all information related to AOPs and any key event (MIE, KE, AO) were downloaded from the AOP-Wiki database on the 22nd of May 2023 (version 2.6; https://aopwiki.org/info_pages/5).

#### 2.1.1 Data set for the gene/protein space exploration

To extract the complete set of genes and proteins involved in existing AOPs, we performed a comprehensive evaluation of the 1371 biological events (MIEs, KEs and AOs) derived from the 403 AOPs contained in the AOP-Wiki database. Considering the inter-species characteristics of AOPs and the collaborative nature of the AOP-Wiki, it was crucial to consider possible redundancies in gene and protein annotations including synonyms and orthologs. To ensure the uniqueness of biological entities and eliminate synonyms, a meticulous manual verification process was conducted (e.g., the NR1I3 receptor is labeled as Androstane receptor, CAR, or NR1I3 depending on the AOP authors). Then, to facilitate data integration and interpretation, as well as to avoid potential conflicts arising from inter-species genes during subsequent enrichment steps, we decided to retain only human genes and map them to their corresponding HGNC gene nomenclature (https://www.genenames.org/) (Seal et al., 2023). For non-human genes, we performed a search for the presence of human orthologs using the HGNC Comparison of Ortholog Predictions (HCOP) tool (https://www.genenames.org/tools/hcop/), aiming to preserve maximum information for subsequent analyses. The complete list of extracted genes and proteins is presented in Supplementary Table S1. Genes mapped to their HGNC symbols were subsequently used for the exploration of biological pathways using Gene Ontology (GO) and for disease associations using DisGeNET (as detailed in Sections 2.2.1 and 2.2.2, respectively).

#### 2.1.2 Data set for the disease space exploration

From the 403 existing AOPs, we extracted 194 unique AOs. In the AOP-Wiki database, some events can be categorized differently depending on the AOP they are involved in, according to the developer the same event can be labeled as either an MIE, a KE or an AO (e.g., event ID 759 ‘*Increased, Kidney failure*’ was labeled as an AO in the AOP ID 33 and 447 while it was labeled as a KE in the AOP ID 377 and 413). No curation was done to assess and count the actual number of AOs scattered in all the AOPs, i.e., data related to AOP-Wiki events is represented as it was in the AOP-Wiki, so that the event ID 759 ‘*Increased, K. failure*’ was counted as occurring twice as an AO. Then, each compiled AO was manually checked by experts to avoid redundancy of information as some may be synonyms. For example, the AO ID 1458 ‘*Pulmonary fibrosis*’ and AO ID 1276 ‘*lung fibrosis*’ were merged into one unique AO. The name chosen among all the synonyms of an AO was arbitrary, for instance, we kept ‘*lung fibrosis*’ in the above example, so that ‘*lung fibrosis*’ encloses the number of occurrences of AO ID 1458 and AO ID 1276. After merging synonyms, a total of 149 AOs were kept for the classification (Supplementary Table S2 sheet 1 *ICD_11 classification* and sheet 6 *synonyms*). These AOs were involved in 379 AOPs among the 403 currently present in the AOP-Wiki, meaning that 24 AOPs did not have any AO events listed.

### 2.2 Mapping the AOPs to the biological space using gene/protein information

#### 2.2.1 Gene and pathways

An overrepresentation analysis (ORA) was performed on 22 May 2023, using the Gene Ontology (GO) database (Ashburner et al., 2000), which classifies genes based on their Cellular Component (CC), Molecular Function (MF), and Biological Process (BP) annotations. The analysis was conducted with the g:Profiler tool (version e109_e.g.,56_p17_1d3191d) (Raudvere et al., 2019) using a Fisher’s one-tailed test (or hypergeometric test). The Benjamini–Hochberg False Discovery Rate (FDR) method was applied to adjust the *p*-values, and an adjusted *p*-value threshold of 0.05 was used to select enriched GO terms. The results for the BP, CC, and MF categories are provided in Supplementary Table S3. To simplify the visualization and interpretation of the ORA results, the ReviGO tool was used (Supek et al., 2011). By applying a clustering algorithm that incorporates semantic similarity measures, ReviGO reduced data redundancy while facilitating the graphical representation of the most relevant terms through multidimensional scaling (MDS).

#### 2.2.2 Genes and diseases

Each selected HGNC symbol was investigated for disease associations using the DisGeNET database, a comprehensive platform containing one of the largest publicly available collections of genes and variants associated with human diseases (Piñero et al., 2020). The purpose of this analysis was to identify potential disease associations and gain further insights into the biological implications of our gene list. We utilized the *disease_enrichment* function from the disgenet2r R package to search for disease associations (Piñero et al., 2020). The results were filtered based on a False Discovery Rate (FDR) threshold of 0.05 to select statistically significant associations. In addition to the disease enrichment analysis, a second analysis was performed using the gene2disease function from the disgenet2r R package, with the list of genes from the AOP and the curated DisGeNET database. This analysis allowed us to explore the relationship between the genes and their corresponding Medical Subject Headings (MeSH) disease classes. A visualization was created to display the associations between the genes and the MeSH disease classes, using a chord diagram to better understand these relationships. To enhance clarity and focus on the most relevant associations, only relationships with greater than 60% involvement were kept in the final visualization.

### 2.3 Mapping of the AOP to the disease space using the adverse outcome information

#### 2.3.1 Disease enrichment

To get an overview of existing data obtained from the AOP-Wiki and to facilitate further analysis, all selected AOs from 2.1.2 were automatically classified using the 11th International Classification of Diseases system (ICD-11) provided by the World Health Organization (https://icd.who.int/browse11/l-m/en ). The classification outcome was further checked and curated by experts within the field of human toxicology. ICD-11 version was launched 1st January 2022 and contains a total of 24 disease categories. To have the most complete overview of the diseases present in the different AOPs already developed, the unclassified AOs using ICD-11 were checked by experts, and, if needed, new classes were created.

#### 2.3.2 Identification of gaps

To avoid any interpretation bias concerning AOPs that have been over- or insufficiently appraised, we caried out a formal statistical analysis to investigate the biological areas that are most studied, and those where research needs to fill in the gaps. With no preconceived assumptions regarding the AOPs distribution from the AOP-Wiki database, ICD-11 categories that were statistically too common or too rare within the AOP space were investigated. Therefore, we first performed a Chi-Square Goodness of Fit (χ^2^ GoF) test (Pearson, 1900). Particularly, we tested whether the distribution of the ICD-11 categories was uniform (H_0_), i.e., whether all categories were equally distributed across existing AOPs in the AOP-Wiki. If there is no discrepancy (i.e., all classes are studied in the same proportion), the full distribution should be uniform. To avoid bias in the χ^2^ GoF test, AOPs belonging to the ‘unclassified’ home-made family class and those belonging to six categories including ‘Conditions related to sexual health’, ‘Factors influencing health status or contact with health services’, ‘Symptoms, signs or clinical findings, not elsewhere classified’, ‘Injury, poisoning or certain other consequences of external causes’, ‘External causes of morbidity or mortality’ and ‘Decrease, Population growth rate’ were removed. Such categories are imprecise and may include terms that are too divergent from each other, in addition to hosting non-humans AOs. They are also not expected to be equally distributed compared to specific categories of a particular disease type. Therefore, the distribution was tested on 304 AOPs spread over 18 classes. Subsequently, we conducted a *post hoc* test to investigate which classes were over- or under-represented. In particular, we computed Haberman’s residuals to determine which classes have contributed the most to the rejection of H_0_, leading to a non-uniform distribution of ICD-11 categories in AOP-Wiki (Agresti, 2007; Sharpe, 2015). These so-called standardized residuals r_i_ were computed for each category according to the following formula (Eq. 1):

$$r_{i}=\frac{\left(observed_{i}\;-\;expected_{i}\right)}{\sqrt{N\times p_{i}\times \left(1-p_{i}\right)}}$$ (1)

where ‘observed _i_’ is the actual number of observed AOPs belonging to a specific class i, ‘expected _i_’ the number of expected AOPs belonging to the class i under the null hypothesis (expected _i_ = 304/18 ≈ 17 ∀ i since H_0_ states that the distribution should be uniform), N the number of AOPs considered in the test (N = 304) and p_i_ the probability that an AOP drawn at random from the 304 considered for the test belongs to the class i under the null hypothesis (p_i_ = 1/18 ∀ i since H_0_ states that the distribution should be uniform). The larger the residual in absolute value, the greater the contribution of the category to the magnitude of the χ^2^ value obtained and the more the category was over- or under-studied (the residual sign indicates the direction of the gap).

### 2.4 Case studies

The three case studies that were prioritized within the PARC project were evaluated in depth by presenting AOPN from existing data in the AOP-Wiki. This identifies the state-of-the art for chosen cases, also giving an overview in which areas future development should take place. These three cases correspond to Immunotoxicity and Non-Genotoxic Carcinogenesis (Case 1), Endocrine and Metabolic Disruption (Case 2), and Developmental and Adult Neurotoxicity (Case 3).

The AOP-DB RDF tool (Martens et al., 2022) was used to extract the data for the subcases with customized queries for each one. Furthermore, key events common to all subcases were connected, when possible, followed by a graphical visualization of the AOP network with Cytoscape v3.10.0 (Shannon et al., 2003).

## 3 Results

### 3.1 Extraction and preparation of the AOP information from the AOP-Wiki database

**FIGURE 2:**
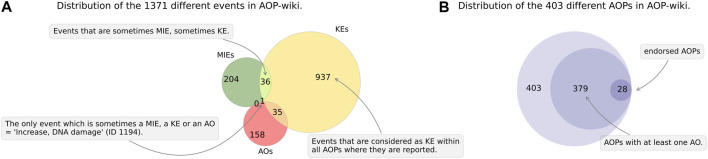
The AOP-Wiki data. Current data related to submitted AOPs (endorsed or not) in the AOP-Wiki database, as of 22 May 2023. **(A)** Green circle: events that are considered as a MIE within all AOPs where they are reported, red circle: events that are considered as AO within all AOPs where they are reported, orange crescent: events that are sometimes KE, sometimes AO. **(B)** Outer circle: total number of AOPs in the AOP-wiki, intermediate circle: number of AOPs with at least one AO, inner circle: total number of OECD-endorsed AOP (‘WPHA/WNT Endorsed’ status) with at least one AO.

From the AOP-Wiki database, we extracted information for 403 different AOPs, that include 1371 unique ID events (Figure 2).

Of the 1371 unique ID events, 204 were labeled as MIEs (i.e., events which are always referenced as MIEs within all AOPs), 937 were labeled as KEs and 158 as AOs. Some other events belonged to several types, as they are defined depending on the AOP they are involved in and were sometimes a MIE or KE (n = 36), KE or AO (n = 35), and even an MIE, KE or AO in one case (e.g., ID 1194‘*Increase, DNA damage*’ which is an MIE in the AOP ID 293, a KE in AOP ID 200 and an AO in the AOP ID 294) (Figure 2A). The existence of such events with different statuses depending on the AOP is a way of extending the concept of AOPs to AOPNs (Knapen et al., 2018). The interconnection between the same event belonging to several AOPs is one of the pillars of the AOP-Wiki philosophy, which seeks to connect AOPs. For example, the event ID 1194 is used about 11 times, which allows it to be frequently cited (all states KE, MIE, AO- Rank 10). Out of the 403 AOPs surveyed, 379 have at least one AO. Among the 379 AOPs with at least 1 AO, only 28 have the ‘WPHA/WNT Endorsed’ status (∼7%) (Figure 2B). The AOP ID 21 ‘*Aryl hydrocarbon receptor activation leading to early life stage mortality, via increased COX-2*’ has this status but did not have any AO reported, bringing the total number of OECD-endorsed AOPs to 29.

### 3.2 Global mapping of the AOP to the biological space using gene information

#### 3.2.1 Data set for the biological space exploration

A total of 245 genes or proteins were manually extracted from the 1371 events contained within the 403 AOPs. After removing duplicates resulting from the collaborative nature of the AOP-Wiki (e.g., see 2.1.1 for NR1I3), a total of 220 unique entities were kept. Then, all 220 genes were mapped to their corresponding symbols to facilitate GO and DisGeNET enrichment analysis. Among these, 149 genes were directly associated with a HGNC gene symbol (represented in green in Supplementary Table S1). Additional 14 entities were manually verified by experts (e.g., when isoform information was missing) and successfully mapped (represented in orange in Supplementary Table S1). However, 57 entities could not be mapped (represented in red in Supplementary Table S1). Some of these genes did not correspond to human genes and lacked a human ortholog (e.g., Vitellogenin), resulting in their exclusion from the enrichment analysis. On the other hand, some genes could not be mapped due to insufficient precision during the AOP development. For instance, among the most frequently unmapped entities, protein families such as Cytokines or Caspases were found in 7 and 6 AOPs, respectively.

Consequently, the total number of genes properly associated with their HGNC symbols has been raised to 163, being the starting point for both GO and DisGeNET analysis.

#### 3.2.2 Gene and pathways exploration

Among the genes prominently represented in our final list, (n = 163), the Angiotensin Converting Enzyme 2 (ACE2) and Aryl Hydrocarbon Receptor (AHR or AhR) are the most frequently mentioned in the AOP-Wiki, appearing in more than 17 distinct AOPs (Supplementary Figure S1). The prominent presence of these proteins in numerous toxicity pathways can be explained by their important biological roles. For example, ACE2, being a multifunctional protein (Turner, 2015), is found in various AOPs related to diverse organs (e.g., pulmonary fibrosis in AOP ID 319, renal dysfunction in AOP ID 384, and cardiac dysfunction in AOP ID 427). Moreover, a wide range of AOPs including ACE2 are related to the SARS-CoV-2 virus (COVID-19) (https://www.ciao-covid.net/aops), which has been extensively studied in recent years (AOP IDs 379, 426, 430, 468), as the virus uses ACE2 as a cellular entry point for infection (Beyerstedt et al., 2021). The AhR is a cytosolic transcription factor that plays a significant role in various cellular processes, including xenobiotic metabolism. It is primarily associated with environmental pollutants, such as dioxins and polycyclic aromatic hydrocarbons. Activation of the AhR is linked to metabolic disorders (e.g., AOP ID 57) and the progression of specific cancer types, which explains its presence in various cancer related AOPs (AOP IDs 416, 417, 420, and 439) (Wang et al., 2020). However, the AhR is also regulated by endogenous compounds including tryptophan metabolites, by microorganisms or ultraviolet light (Fritsche et al., 2007; Rannug, 2022). This will likely increase further the number of AOPs in which it is involved.

**FIGURE 3:**
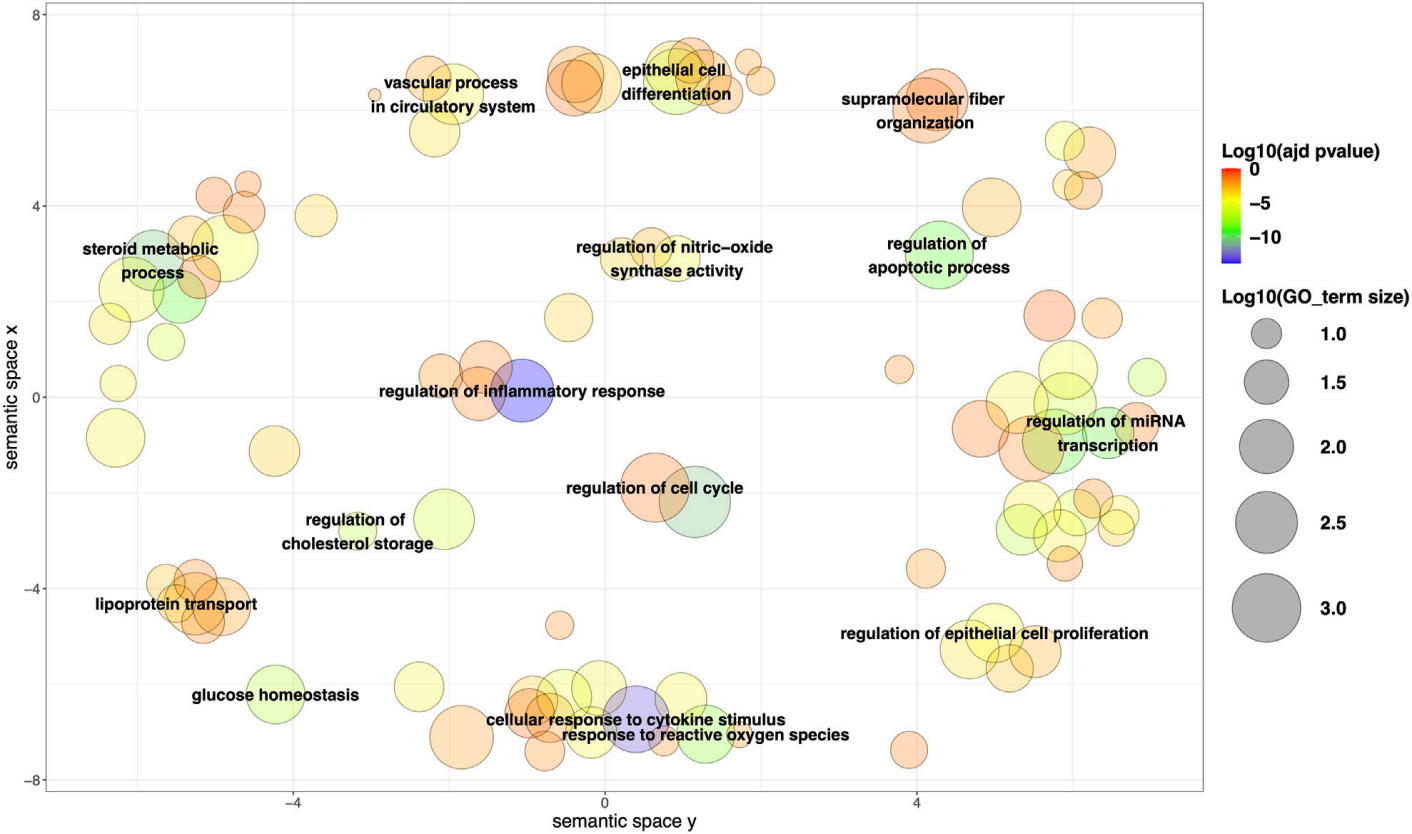
Representation of the most relevant biological process (BP) after clustering based on semantic similarity and multidimensional scaling using ReviGO. The size of the points is proportional to the size of each BP term, while the color represents the adjusted p-value (log10). The following settings were used: Default settings; Work With (Homo Sapiens); Resulting list (Tiny).

After the GO enrichment of the 163 mapped genes, a total of 963 significant terms (adjusted *p*-value <0.05) were obtained for BP, 87 for MF, and 60 for CC (Supplementary Table S3). Among the most relevant BPs identified using ReviGO, a substantial representation of metabolism-related processes was observed (e.g., glucose homeostasis, regulation of cholesterol storage, steroid metabolic process, lipoprotein transport). Additionally, there is a notable presence of BPs commonly associated with cancer, such as cellular regulation (e.g., cell differentiation, regulation of apoptotic process, regulation of miRNA transcription, regulation of cell proliferation), as well as processes related to oxidative stress and inflammation (Figure 3). Among the most frequently observed genes in metabolism-related processes, examples include AhR and LXR in AOPs associated with steatosis (e.g., AOP IDs 34, 57, 58 and 232), PPARα in AOPs describing lipid metabolism alterations (e.g., AOP ID 166), and ERα in an AOP related to obesity (AOP ID 493). For processes involved in tumor progression (cellular regulation, oxidative stress, inflammation, etc.), numerous genes are implicated in tumor progression (e.g., ERs contribute to disruptions in proliferation, apoptosis, and/or oxidative stress in AOP IDs 167 and 200). AhR activation leads to increased inflammation and/or apoptosis in AOP IDs 419 and 439, disruptions in cell proliferation in AOP ID 420, and the accumulation of ROS in AOP IDs 418 and 420). This trend was confirmed by the results obtained for MF. Among the most relevant enrichment results, we particularly found ‘cholesterol binding’, ‘lipid binding’, ‘cytokine receptor binding’, ‘oxidoreductase activity’, and ‘molecular function regulator activity’, which align with the identified BPs related to metabolism and cellular regulation (Supplementary Figure S2). Additionally, there are results such as ‘nuclear receptor activity’ and ‘signaling receptor activator activity’, which correspond to genes and proteins frequently found across all AOPs, such as steroid receptors (androgens and estrogens), LXRs, PXR, CAR, and others.

#### 3.2.3 Gene and disease exploration

To complement the work on biological pathways performed above, a disease enrichment analysis was conducted using the DisGeNET database, based on the 163 mapped genes (Supplementary Table S1). The goal of this analysis was to gain further insight into the biological implications of the gene list to expand our understanding of disease associations with AOPs. For the first analysis, a disease enrichment analysis was performed, and a total of 1215 significantly enriched diseases (FDR <0.05) were identified. These 1215 diseases group into 165 unique DisGeNET disease classes, based on the MeSH hierarchy. The complete list of significantly enriched diseases can be found in Supplementary Table S4, and Supplementary Figure S3. In the second analysis, the *gene2disease* function was used to explore gene-disease associations and visualize the relationships between the genes in the list and the MeSH disease classes they are associated with. Gene-disease associations were computed individually, yielding percentages to measure gene involvement within specific disease classes relative to overall disease associations. Subsequent investigation focused on associations where gene involvement surpassed a 60% threshold, emphasizing pronounced connections between genes and specific diseases. The resulting chord diagram (Supplementary Figure S4) effectively illustrates the associations between DisGeNET diseases and HGNC genes. Interestingly, the genes that are strongly connected with the DisGeNET disease classes are different from those that are most frequently found in the AOPs. This suggests that many of the DisGeNET disease classes represented in the gene-disease associations are not adequately captured by the existing AOPs, highlighting potential areas where the AOP framework could benefit from further development. One reason for this might be taxa specificities. For example, AOP ID 41 ‘Sustained AhR Activation leading to Rodent Liver Tumours’ is a very well defined AOP established in rodents which is not directly transferable to the human system. After applying the 60% threshold filter, only 8 genes were related to 6 disease classes (Supplementary Figure S4). This highlights the value of performing a disease enrichment analysis to uncover novel associations and better understand the links between the AOPs and various diseases. By identifying diseases that are significantly enriched in relation to the genes involved in AOPs, we gain insights into potential AOs that may not have been previously considered. This information can contribute to a more comprehensive understanding of the adverse effects associated with the molecular events in AOPs and aid in refining risk assessment and mitigation strategies.

### 3.3 Global mapping of the AOP to the disease space using AO information

#### 3.3.1 Preliminary feedback for MIEs, KEs and AOs

The most studied MIEs are both the activation of the AhR (ID 18) and a deposition of energy (ID 1686) with 17 occurrences each, followed by ‘*Binding to ACE2*’ (15 occurrences) (Supplementary Figure S5). As AhR and ACE2 are proteins, this was consistent with the results looking at the most studied proteins (see 3.2.2), suggesting here that the majority of MIEs involve a deregulation of a protein (through activation or inhibition). Indeed, among the 30 most common MIEs identified, 21 that were directly protein-based.

For the KEs, the event ‘*Thyroxine (T4) in serum, Decreased*’ (ID 281) is the most studied, with 24 occurrences followed by ‘*Thyroid hormone synthesis, Decreased’* (ID 277; 18 occurrences) and ‘*Cell injury/death*’ (ID 55; 17 occurrences) (Supplementary Figure S6). Unsurprisingly, there was a greater disparity of events which can be understood because an AOP is supposed to have only one (or two or even three) MIE(s) but several KEs at various organizational levels. ACE2-related events such as ‘*SARS-Cov-2 cell entry*’ (ninth place, 10 occurrences) can still be found among the top 30 kEs as well as cancer-related and inflammation-related events such as ‘*Inadequate DNA repair*’ (ninth place, 10 occurrences), ‘*Increase, Cell Proliferatio*n’ (11th place, 8 occurrences) or ‘*Induction, Epithelial Mesenchymal Transition*’ (12th place, 7 occurrences) and ‘*Increased, secretion of proinflammatory mediators*’ (eighth place, 11 occurrences) or ‘*Increased, recruitment of inflammatory cells*’ (11th place, 8 occurrences) respectively.

The distribution of the 30 most common AOs is shown in Supplementary Figure S7 (see Supplementary Table S2 sheet 4 *full_distribution AOs* for the complete list). It appears that the event ID 360 ‘*Decrease Population Growth Rate*’ is the most represented in the AOP-Wiki (57 occurrences), followed by ‘*Increase Mortality*’ (37 occurrences) and ‘*Death/Failure, Colony*’ (21 occurrences), thus reflecting the field of ecotoxicology. In non-mammalian species, decreases in population growth rates are strongly causally linked with disorders related to reproduction since 27 out of 57 AOPs hosting this AO co-mentioned terms such as ‘*Impairment of reproductive capacity*’, ‘*Decreased fecundity*’ or ‘*Reduction, Cumulative fecundity or spawning*’. That the AOP concept was initially established for ecotoxicological questions (Ankley et al., 2010) might explain the overrepresentation of ecotoxicological AOs. Among the top 30 AOs, we also saw 2 terms related to cancer (‘*Lung cancer*’, 5 occurrences; ‘*N/A, Breast Cancer*’, 4 occurrences) which represent the two most common cancers worldwide (Mattiuzzi and Lippi, 2019). We observed the presence of diseases related to metabolism (‘*Increased, Liver Steatosis*’, 9 occurrences; ‘*Occurrence, Kidney toxicity*’, 5 occurrences) or neurological type (‘*Cognitive Function, Decreased*’, 12 occurrences; ‘*Occurrence, Epileptic seizure*’, 8 occurrences). These most represented AOs are in line with the GO enrichment of the 163 mapped genes since they fit with BP related to cancer and metabolism.

Here we would like to bring to the reader’s attention that the frequency distribution of KEs published in the AOP-Wiki, similar to the general published literature, does not represent their importance for human diseases due to funding and publication bias caused by the ‘streetlight effect’ (Newquist et al., 2015; Evans, 2020).

#### 3.3.2 Mapping the AOPs to the disease space

**FIGURE 4:**
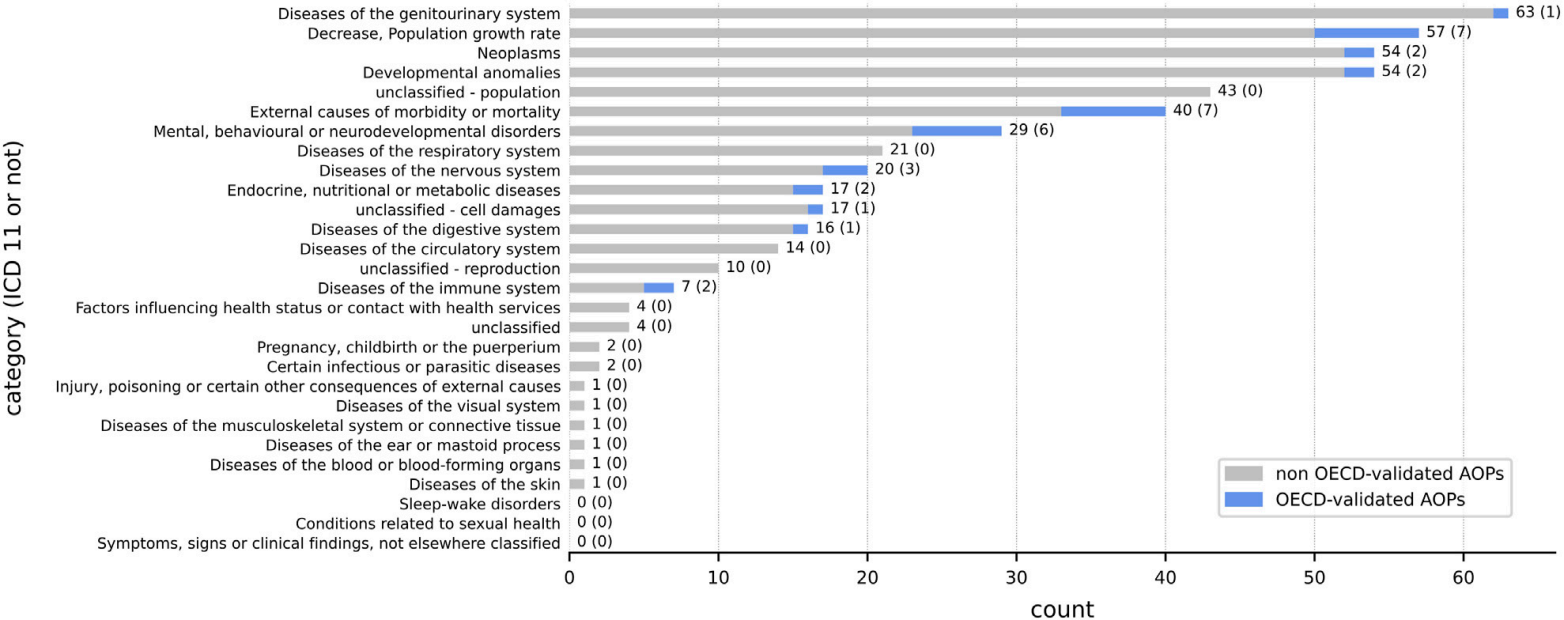
Classification of the 379 AOPs that mention at least one AO using the ICD-11 system. The classification was performed using the AO information associated with the existing AOP in the AOP-Wiki database. The number at the end of each row represents the total number of AOPs (endorsed or not) while the number in brackets represents the number of AOPs with the ‘WPHA/WNT Endorsed’ status. The sum does not equal 379 because an AOP can be classified into several categories (1 AOP ⇏ 1 AO). The associated OECD-endorsed AOP IDs are listed in Supplementary Table S2 sheet 3 clustering_AOPs.

Among the 149 AOs identified in Section 2.1.2, we were able to classify only 127 AOs using the ICD-11 classification. To have the most complete overview of the diseases present in the different AOPs already developed, the 22 unclassified AOs using ICD-11 were checked by experts, and when needed new classes were created. A total of four new classes were proposed: i) one related to reproduction, ii) one related to population, iii) one for cell damages and iv) a last one for all the AOs that were not fitting within all these classes (Figure 4) (Supplementary Table S2 sheet 1 *ICD_11 classification*). We noticed that some AOPs were connected to several AOs. This led to a total of 480 AOs disseminated in 379 AOPs and AOPs with more than one AO were classified into several categories. For example, the AOP ID 402 had 2 AOs including ‘*Periventricular heterotopia formation*’ and ‘*Occurrence, Epileptic seizure*’ and has been thus classified in the ‘Developmental anomalies’ and ‘Diseases of the nervous system’ ICD-11 categories (Supplementary Table S2 sheet 5 *AOs_in_AOPs*). In the present study, AOPs without any AO (n = 24) were not integrated in the classification.

It appears that diseases of the genitourinary system are the most studied, followed by the AO ‘Decrease, Population Growth Rate’, neoplasms and developmental anomalies. The AO ‘Decrease, Population Growth Rate’ is so widely studied that it is present in more AOPs than any of the ICD-11 classes (excluding ‘Diseases of the genitourinary system’) that yet group together several AOs. It is noticeable that only 7 AOPs refer to diseases of the immune system but 2 out of 7 of them are OECD-endorsed while 21 AOPs refer to diseases of the respiratory system but 0 out of 21 are OECD-endorsed (Figure 4).

As statistical tests are carried out on human diseases, the pseudo-category ‘*Decrease, Population Growth Rate*’ (n = 57 AOPs) has been subsequently ruled out, in addition to the considerations set out in point 2.3.2. Moreover, AOPs belonging to classes ‘unclassified’ (n = 74), ‘Conditions related to sexual health’ (n = 0), ‘Factors influencing health status or contact with health services’ (n = 4), ‘Symptoms, signs or clinical findings, not elsewhere classified’ (n = 0), ‘Injury, poisoning or certain other consequences of external causes’ (n = 1) and ‘External causes of morbidity or mortality’ (n = 40) have also been removed in line with the hypothesis adopted (2.3.2). Finally, statistical tests were carried out on the 304 AOPs spread over 18 classes that fit the assumptions.

### 3.4 Gaps

**FIGURE 5:**
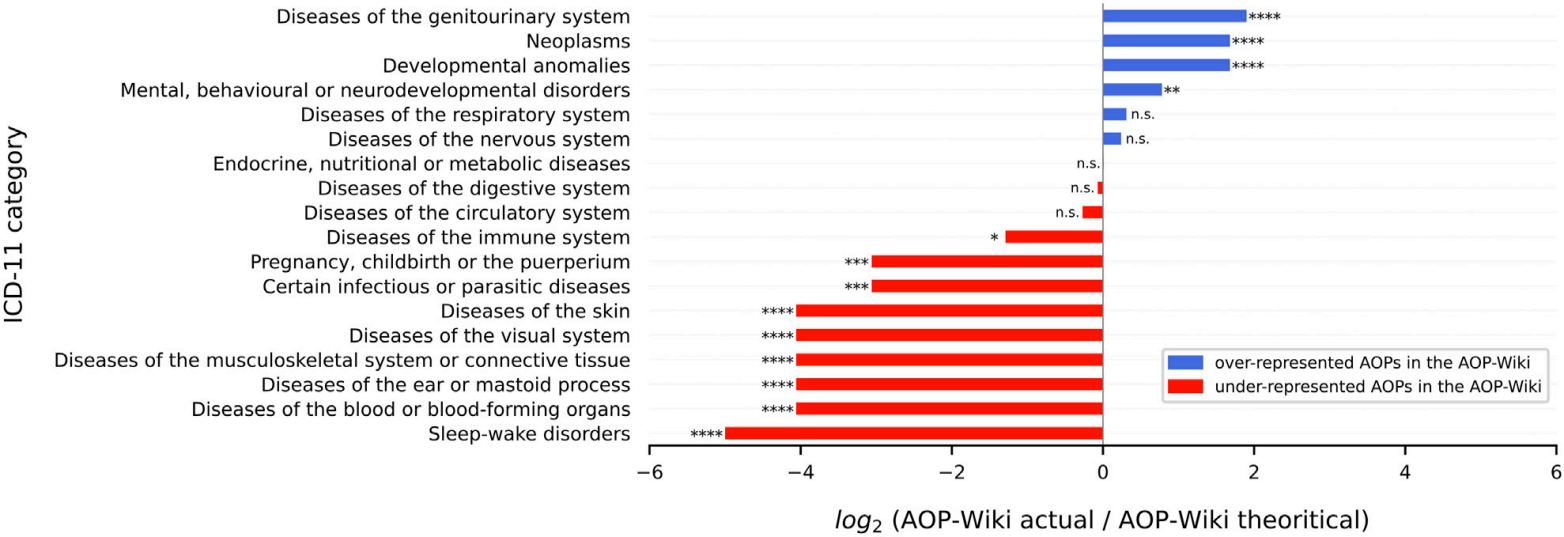
Imbalance in the representation of the AOPs in AOP-Wiki using the ICD-11 disease classification. The quantity plotted here is the binary logarithm of the ratio between the actual number of AOPs and theoretical AOPs in AOP-Wiki. This representation was chosen for convenience as it is clearer than the residuals which are more subtle. For instance, the value of 1.90 ≈ 2 for diseases of the genitourinary system indicates that there are about 22 = 4 as many AOPs in this category as there should be if the distribution of all categories was uniform. A value of 0 means that the ratio is equal to 1, i.e., the number of actual AOPs perfectly matches the number of theoretical AOPs. When there were exactly 0 AOPs in a category (‘Sleep-wake disorders’), we arbitrarily set the log2 value to −5 to avoid forbidden values, as log2 (0) is not defined. However, the p-value computed is associated with the residuals and not with the log2 value of the ratio. n. s = p ≥ 0.05, * = 0.01 ≤ p < 0.05, ** = 0.001 ≤ p < 0.01, *** = 0.0001 ≤ p < 0.001, **** = p < 0.0001.

To identify gaps (i.e., AOPs that were not yet developed or poorly developed for some disease categories), statistical analysis was performed using the previous classification with ICD-11. A χ^2^ GoF test followed by a *post hoc* test (residual analysis) was performed to investigate which classes were over- or under-represented (Figure 5; Supplementary Table S2 sheet 7 *gaps*) according to the presumed distribution (2.3.2).

The distribution of the 18 ICD-11 categories in AOP-Wiki considered for the χ^2^ GoF test was widely skewed (χ^2^ = 423.46; *p* = 2.28e-79). On the one hand, the *post hoc* test (residual analysis) highlighted that 3 classes were largely overstudied including ‘*diseases of the genitourinary system*’, ‘*neoplasms*’ and ‘*developmental anomalies*’ (*p* < 0.0001). On the other hand, it highlighted that most of the other classes were under-studied, which reflects the large disparity in the study of AOPs. Currently, there are only 5 categories (*p* > 0.05) that are correctly distributed (under the null hypothesis H_0_ that the distribution should be uniform). For instance, the residual value of the ‘*Endocrine, nutritional, and metabolic diseases*’ class is, following the Eq. 1 in 2.3.2:

$$r_{endocrine,nutritional\;or\;metabolic\;disorders}=\frac{\left(17\;-\;16.89\right)}{\sqrt{304\times \frac{1}{18}\times \left(1\;-\;\frac{1}{18}\right)}}\approx 0,03$$

where 17 is the actual number of observed AOPs belonging to the ‘*Endocrine, nutritional and metabolic diseases*’ class, 16.89 the number of expected AOPs belonging to this class under the null hypothesis (=304/18 ≈ 16.89 since H_0_ states that the distribution should be uniform), 304 the number of AOPs considered in the test and 1/18 the probability that an AOP drawn at random from the 304 considered for the test belongs to the ‘*Endocrine, nutritional and metabolic diseases*’ class under the null hypothesis.

Therefore, the highest value in the dataset showed that the ‘*diseases of the genitourinary system*’ class contributed the most to the rejection of H_0_ and is the furthest from the uniform distribution. Particularly, r = 11.54 > 0 (*p* = 8.29 × 10^−31^) so this class was over-represented in the AOP-Wiki. Moreover, results showed a concordance with the 25 most significant diseases found with the curated DisGeNET database (Supplementary Figure S3), especially regarding cancer (neoplasms) terms. Not only does cancer have a prominent place in the AOP-Wiki database with the highest number of AOs referring to it (n = 25, Supplementary Figure S8, Supplementary Table S2 sheet 2 *clustering_AOs*) and the second class the most studied (r = 9.29; *p* = 1.54 × 10^−20^), but it is also the field that gathers the highest number of terms (MIE, KE or AO) as shown by the analysis with the CURATED DisGeNET database (Supplementary Figure S3) in addition to the GO enrichment analysis (see 3.2.2).

However, a parent category that fits to its theoretical number of AOPs may have a non-uniform distribution within its daughter categories. As an example, for the ‘*Endocrine, nutritional, and metabolic diseases*’ class which is neither over- nor under-studied (*p* = 0.98), we noticed that within it the distribution was not uniform (Supplementary Figure S9). The most studied pathologies are those related to the liver such as ‘*liver steatosis*’ and ‘*steatohepatitis*’ which represent almost two-thirds of the parent class. Besides, there are only a few AOPs that cover ‘*Endocrine, nutritional, and metabolic diseases*’ excluding the liver. Particularly, only a minority of AOPs report diseases such as obesity and diabetes which contrasts the numerous studies in the literature outside the AOP-Wiki framework that deals with the presence of obesogenic and diabetogenic substances in our environment (Wolf et al., 2019; Lam et al., 2023).

### 3.5 Cases studies

To shed light on how different AOPs are represented within the AOP-Wiki, we explored more precisely three case studies that were prioritized within the PARC project. These three cases correspond to Immunotoxicity and Non-Genotoxic Carcinogenesis (Case 1), Endocrine and Metabolic Disruption (Case 2), and Developmental and Adult Neurotoxicity (Case 3).

#### 3.5.1 Case study 1: Representation of immunotoxicity and non-genotoxic carcinogenesis AOPs in the AOP-Wiki and their links to human health

**FIGURE 6:**
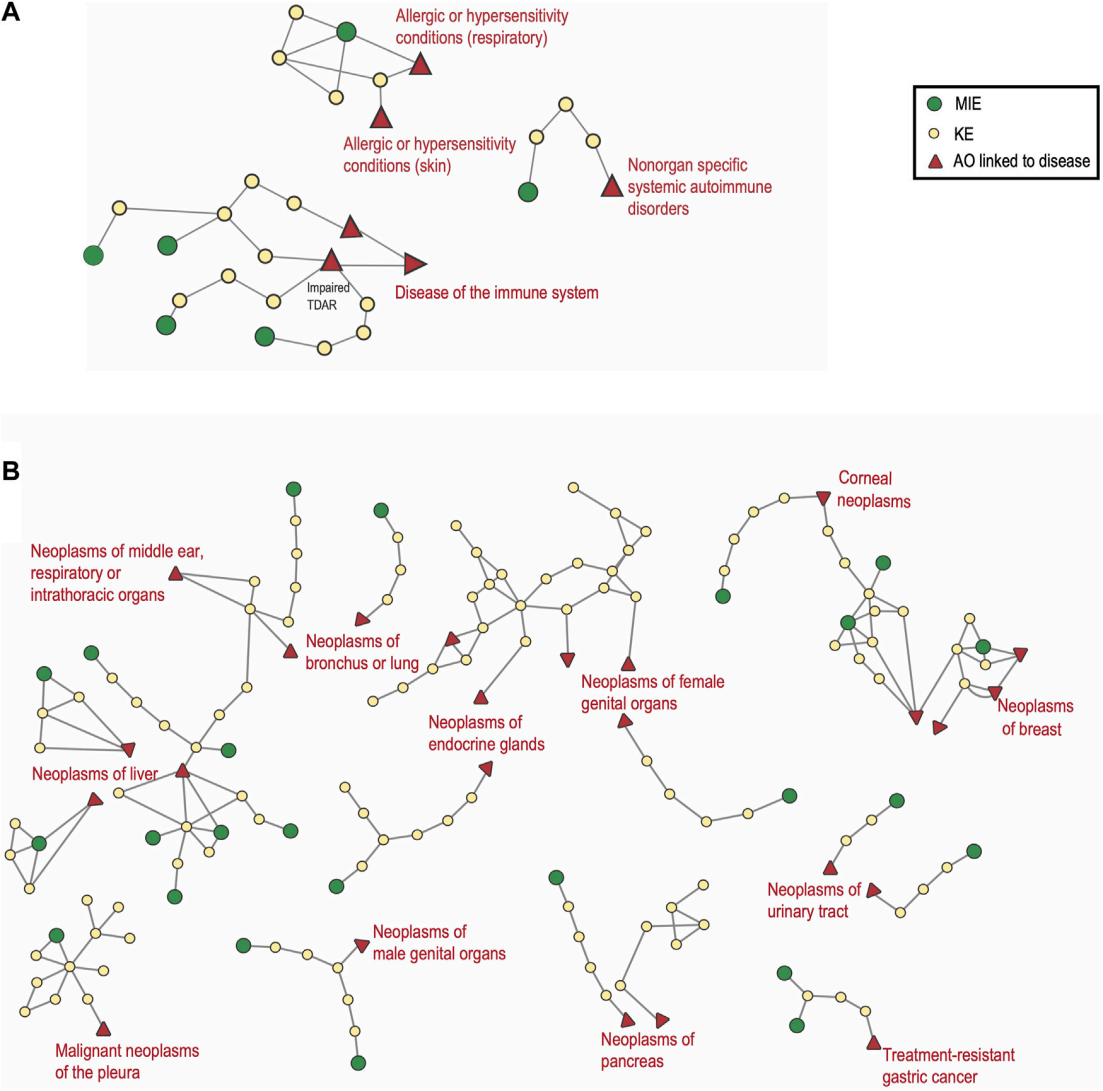
Adverse Outcome Pathway Networks for Immunotoxicity and for Non-Genotoxic Carcinogens. **(A)** covers AOPs related to immunosuppression, sensitization and autoimmunity made up of 4, 2 and one AOP respectively. For the AO Impaired T-cell dependent antibody response “TDAR”, the three AO synonyms were merged. **(B)** 31 AOPs linked to non-genotoxic carcinogenesis are displayed in this AOPN. In both A and B, the relevant ICD-11 disease category is annotated in red. Non-mammalian and empty AOPs were not included.

In the context of immunotoxicity, human health is influenced by four principal processes: hypersensitivity, autoimmunity, immunostimulation, and immunosuppression. Immunotoxic compounds may directly trigger responses towards themselves, suppress, promote and/or aggravate responses to self-antigens/allergens/infectious antigens, and modify disease risk. Actions may be mediated by affecting the intensity of immune responses (insufficient or exaggerated response or duration) or by modulating the immune system leading to inappropriate responses. As mentioned above, AOPs representing immunotoxicity endpoints are currently under-represented in AOP-Wiki (r = −2.48, *p* = 0.013, Figure 5). Nine AOPs representing hypersensitivity, autoimmunity and immunosuppression were identified in the AOP-Wiki. Two AOPs were related to hypersensitivity, one to autoimmunity and six to immunosuppression. Two of the six immunosuppressive AOPs (AOP IDs 84 and 85) have honeybee colony failure as their AOs. Therefore, an AOPN based on the seven AOPs with taxonomic applicability to mammals was constructed (Figure 6A).

For immunosuppression, three complete and one less complete AOP were identified in the AOP-Wiki (AOP IDs 315, 154, 277 and 14, respectively), of which one is endorsed (AOP ID 154; *Inhibition of Calcineurin Activity Leading to Impaired T-Cell Dependent Antibody Response*). Four different MIEs are represented (*Inhibition of calcineurin activity*; *Inhibition of JAK3*; *Impaired IL-1R1 signaling*; *Activation of Glucocorticoid Receptor*) all acting at the level of the T-cell. Two of the AOPs have inhibition of NF-κB as a common KE and three of the AOPs converge on the same AO, Impaired T-cell dependent antibody response (‘TDAR`) for which there are three AO-synonyms used (Supplementary Table S2 sheet 6 *synonyms*). Immunosuppression can be linked to an increased risk of infection as well as an increased risk for certain cancers (World Health Organization and Safety, 2012). In the regulatory setting, the TDAR *in vivo* assay is used as an indicator of immunotoxicity, e.g., in the extended one-generation reproductive study (EOGRTS, OECD TG 443) in which a developmental immunotoxicity cohort may be included if triggered by a concern. TDAR assays provide a functional readout reflecting the joint activities of innate and adaptive cells (Plitnick and Herzyk, 2010). An impaired TDAR is used as a functional marker of immunosuppression. All four AOPs have similar late KEs addressing suppression of lymphocyte/T-cell activity and they all represent well-characterized mechanisms of actions (MIEs and KEs) for different classes of immunosuppressive drugs. The available AOPs for immunosuppression address effects on adaptive immunity and can be mapped to the main ICD-11 category ‘Diseases of the immune system’. None of the AOPs for immunosuppression include KEs related to the involvement of the innate immune system, cell-mediated immunity or direct effects on B-cell populations. There is also a need for further development of AOP ID 14, which covers Glucocorticoid Receptor Activation, and the further inclusion of additional MIEs of relevance for environmental factors such as the suppression mediated by AhR or by mTOR inhibitors. The immunosuppressive effects mediated by AhR signaling and the interaction with estrogen have also been identified as a cross-cutting priority for further work in case study 2.

Immune stimulation is most understood to involve exaggerated or prolonged inflammation and/or tissue damage, which underlies a wide range of pathological processes and is linked to numerous diseases, including states of hypersensitivity/allergies and autoimmunity. Many immune system-related diseases arising in nonlymphoid tissues are categorized in the ICD-11 based on the target tissue (e.g., skin, lung) rather than as a ‘disease of the immune system’. One example is AOP ID 313 ‘*Stimulation of TLR7/8 in dendritic cells leading to Psoriatic skin disease*’, an inflammatory skin disorder categorized under ‘Diseases of the skin’. Autoimmune diseases result from a harmful immune response directed against the body’s own organs, tissues, and cells. More than 80 autoimmune diseases are recognized, being either systemic or affecting specific organs. Among the more common are type 1 diabetes, rheumatoid arthritis, systemic lupus erythematosus, inflammatory bowel disease and autoimmune thyroid diseases. Environmental factors are linked to autoimmune disease etiology (Parks et al., 2014; Conrad et al., 2023). However, the specific mechanisms leading to autoimmune diseases and the effects of environmental exposures remain for the most part poorly understood. In the AOP-Wiki, one AOP for autoimmune disease was identified; the AOP ID 314 ‘*Binding to estrogen receptor (ER)-α in immune cells leading to exacerbation of systemic lupus erythematosus (SLE)*’. SLE is categorized in the ICD-11 under ‘Diseases of the immune system 04’ and the subcategory ‘Nonorgan specific systemic autoimmune disorders’. Several autoimmune diseases are increasing in incidence, like type 1 diabetes, but are currently not represented in the AOP-Wiki.

Two AOPs related to the immune system disease subcategory allergic, or hypersensitivity conditions are represented in AOP-Wiki: AOP ID 40 ‘*Covalent Protein binding leading to Skin Sensitisation*’ and AOP ID 39 ‘*Covalent Binding, Protein, leading to Increase, Allergic Respiratory Hypersensitivity Response*’. The status of the latter is still under development, while AOP ID 40 is considered in chemical regulation (OECD, 2027). Both AOP IDs 39 and 40 describe pathways for responses directed towards the compound itself and share the MIE ‘*Covalent Binding, Protein*’ (MIE ID 396) as well as the 2 KEs: ‘*Activation, Dendritic Cells*’ (KE ID 398) and ‘*Activation/Proliferation, T-cells’* (KE ID 272) (Supplementary Table S5 sheet 1 AOP IDs 39 & 40). Certainly, there are organ-specific differences to consider in the sensitization processes. However, aspects like the secretion of inflammatory mediators and activation of epithelial cells play an important role in both disease processes, thus there may be more mechanistic overlap than can be extracted from a comparison based only on the terminology of the events. Furthermore, there is increasing evidence that the route of exposure does not necessarily restrict itself to the same target organ, with abnormal regulation of immune cell responses playing an important role (Kimber et al., 2011). No AOPs for adjuvant or allergy-promoting processes were identified.

Considerably more work will need to be done to cover immunotoxicity endpoints in sufficient depth (develop AOPs fully) as well as breadth. In particular, developmental immunotoxicity (DIT), which is a regulatory toxicity endpoint of high concern, is currently not represented in the AOP-Wiki. AOPs and (non-animal) test methods targeting the sensitivities of the developing immune system need to be developed to specifically support DIT evaluation as well as other immunotoxicity evaluations. In addition, the important linkages between impaired immune function and the two other focus areas of PARC, endocrine and metabolic disruption, and neurotoxicity, are currently poorly represented in the AOP-Wiki.

Chronic, low-grade inflammation is a major driver and mode of action of non-genotoxic carcinogens (NGTxC). Notably, immune evasion of cancer cells and inflammation make up the two immune-related events that are part of the well-established 10 hallmarks of cancer, described by Hanahan and Weinberg (2011). Within the AOP-Wiki, 45 AOPs, 13 of which remain poorly developed, have been identified that can be linked to NGTxC (Supplementary Table S5 sheet 2 NGTxC AOPs). These include AOPs with and without inflammation as a key event and are linked to the ICD-11 category ‘*Neoplasms* (02)’. As shown in Figure 5, neoplasms feature prominently in the AOP-Wiki (r = 9.29; *p* = 1.54 × 10^−20^). In Figure 6B, 32 of the more developed AOPs have been represented in an AOPN, with their links to the relevant ICD-11 disease subcategory. Lung, liver, and breast cancers are the ones represented by the highest number of AOPs and AhR activation is the most prevalent MIE. This is also reflected by the findings in section 3.2.2, above, which indicates that AhR signaling appears in more than 17 AOPs overall, which alongside ACE2, makes them the most frequent genes/proteins in the AOP-Wiki. Three hub key events have been proposed for the representation of inflammation in AOP networks; ‘tissue resident cell activation’, ‘increased pro-inflammatory mediators’, and ‘leukocyte recruitment/activation’ (Villeneuve et al., 2018b). Eight of the NGTxC AOPs included in the AOPN include inflammation as a KE or a modifying factor.

As cancer has a high contribution to the global burden of disease, further developing AOPs for NGTxC is needed to better predict the contribution of chemical and physical factors to cancer risks. Beyond further developing AOPs, Audebert et al. (2023) also describe the new experimental methods and NAMs that are being developed within the PARC project to map KEs and identify NGTxCs. Although cancer as an AO is well represented in the AOP-Wiki, several of the neoplasms with a presumed environmental or lifestyle component in the human population are not well represented, e.g., testicular seminomas, prostate cancer, colorectal cancer and skin cancer.

Several ongoing projects, including the Aspis cluster (https://aspis-cluster.eu/), the Eurion cluster (https://eurion-cluster.eu/), the EHEN network (https://www.humanexposome.eu/), the CAAT-led working group for alternatives to *in vivo* DIT Testing (https://caat.jhsph.edu/programs/DIT/), as well as PARC, are likely to deliver data and/or test methods (NAMs) that will be suitable to support further development of AOPs for immunotoxicity and NGTxC including the role of lifestyle and environmentally induced chronic inflammation in the promotion of disease.

#### 3.5.2 Case study 2: Representation of AOPs leading to endocrine and metabolic diseases in the AOP-Wiki

This case study focuses on the five current priority areas for AOP development related to endocrine and metabolic disruption in PARC: (i) thyroid hormone system disruption (THSD), (ii) disrupted androgen receptor (AR) signaling, (iii) disrupted estrogen receptor (ER) signaling, (iv) disrupted steroidogenesis, and (v) metabolic disruption. Across these priority areas, 70 AOPs are currently available in the AOP-Wiki. Ten of those AOPs have been endorsed by the OECD WNT/WPHA: AOP ID 6 describing antagonist binding to PPARα leading to body-weight loss, AOP IDs 23 and 25 linking androgen receptor agonism and aromatase inhibition respectively to reproductive dysfunction, AOP ID 42 linking thyroperoxidase (TPO) inhibition to decreased cognitive function, AOP ID 54 linking Na^+^/I^−^ symporter (NIS) inhibition to learning and memory impairment, and AOP IDs 155–159 linking thyroperoxidase and deiodinase inhibition to impaired swim bladder inflation in fish. The latter 7 AOPs are all related to THSD, reflecting a recent and strong international research focus in that area. AOP IDs 42 and 54 lead to developmental neurotoxicity and are relevant in the context of Case study 3 as well.

**FIGURE 7:**
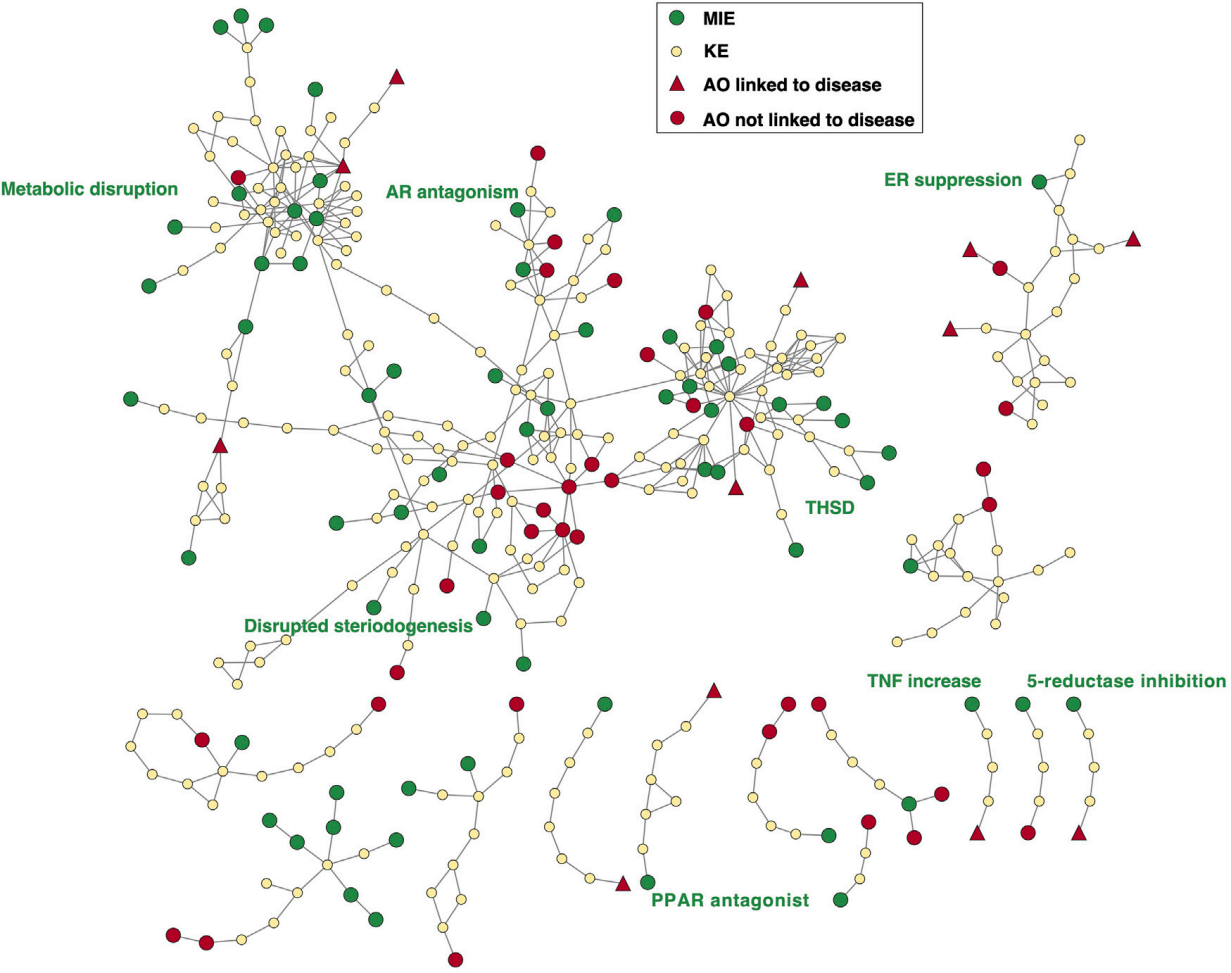
Adverse Outcome Pathway Network consisting of 70 AOPs describing endocrine and metabolic disruption. For each AO linked to a disease, the ICD disease subcategory is shown as a label in red. Labels in green are used to situate the broad areas of endocrine and metabolic disruption in the AOPN. THSD = thyroid hormone system disruption.

An AOPN was constructed that includes all 70 AOPs (Figure 7). Since in this area efforts are being made to bridge the gap between environmental and human health and investigate whether non-mammalian models can be used for informing on human health, we opted to include both AOPs that were initially developed for human health and those initially developed for environmental health. Also, because many of these AOPs had already been curated, we were able to include both complete and incomplete AOPs in this case study to provide a broader picture. Figure 7 shows that most AOPs together form one large AOP network connecting different areas of endocrine and metabolic disruption. While the THSD AOPN has recently been curated to improve the connectivity among AOPs (Haigis et al., 2023), curation may be needed in other parts of the AOP network to reduce duplication and increase connectivity, e.g., to reflect crosstalk between endocrine axes. In the THSD area of the AOP network, hub key events, mostly representing alterations in hormone levels, can be identified where AOPs with multiple MIEs converge and subsequently diverge towards various AOs. In order to investigate the current coverage of endocrine and metabolic diseases by existing AOPs, the ICD-11 category ‘*Endocrine, nutritional or metabolic diseases*’ was identified as the most relevant category for this case study in a first step. Fifteen AOPs have been identified that lead to AOs mapped to diseases in ICD-11, including liver steatosis, steatohepatitis, obesity, decreased body weight and diabetes. Statistically, this disease category was found to be not over- nor under-represented in the AOP-Wiki (r = 0.03; *p* = 0.976; Figure 5). However, as discussed in section 3.4, the focus of AOP developers on diseases such as obesity and diabetes has been relatively limited in general, while the scientific evidence supporting the diabetogenic and obesogenic potential of, for example, pesticides has significantly grown in recent years (Heindel and Blumberg, 2019; Wei et al., 2023). In the broader perspective of diseases related to an organism’s energy metabolism, addressing this gap in AOP development is relevant for both human and environmental health: in humans, adverse health effects largely depend on the dietary context (e.g., a high-caloric, high-fat and high-sugar western diet) while in wildlife, where caloric intake is typically limited, any disruption of energy metabolism may have important consequences for supporting survival, growth and reproduction.

In a second step, which in general was aimed at manually searching for additional AOPs leading to endocrine diseases (i.e., diseases of endocrine organs) that are not captured in the ICD-11 category, we therefore explicitly included AOPs related to metabolic diseases as well. AOPs that have an EDC mechanism but do not have an endocrine or metabolic disease identified as the adverse outcome (e.g., developmental neurotoxicity, impaired fertility, impaired development, hepatocellular carcinoma, endometrial carcinoma, or breast cancer) were not selected for our analysis. Manual identification of those adverse outcomes in the AOPN that could be mapped to the ICD classification system showed that in fact 23 out of 70 AOPs were linked to endocrine diseases (See Supplementary Table S6). Many of these additionally identified AOPs lead to neoplasms of endocrine-active organs (e.g., thyroid, male and female genital organs) which are part of the disease class ‘*Neoplasms*’ which is over-represented in the AOP-Wiki (See section 3.4), presumably due to a historical focus on cancers with a toxicological basis. These paths are also relevant in the context of Case study 1.

Because hormones are involved in regulating almost all aspects of normal development and physiology, Case study 2 differs from the other two case studies in the fact that endocrine and metabolic disruption in an AOP context could be considered as encompassing all pathways leading to endocrine and metabolic diseases on the one hand, or all endocrine mechanisms leading to any adverse health effects (i.e., not restricted to endocrine diseases) on the other hand. As described above, we opted to only focus on endocrine and metabolic diseases for the purpose of the present study. There are, however, some gray areas with respect to linkage between EDC exposure, EDC mechanisms and endocrine diseases. One example is impaired male fertility where the Testicular Dysgenesis Syndrome (TDS) links male reproductive disorders like cryptorchidism, hypospadia and some types of testicular cancer (at least partly) to chemical exposure during sensitive developmental stages (Skakkebaek et al., 2016; Skakkebæk et al., 2022).

Although important advances have been made in the development of AOPs for endocrine and metabolic disruption and a considerable number of AOPs describe mechanisms leading to endocrine and metabolic diseases, several gaps remain to be addressed. From a regulatory perspective, an important consideration in this context is whether the currently available AOPs and AOP network provides sufficient coverage of the overall toxicological space that is relevant to the assessment of endocrine disrupting chemicals, or whether certain important toxicological mechanisms are not yet captured at all. In this context, several AOP development priorities for endocrine and metabolic disruption have been identified in the PARC project. A priority relates to linkages between THSD and metabolic disruption. It is recognized that while some metabolic outcomes such as liver steatosis have received considerable attention, linkages to hormone imbalance and especially thyroid hormone imbalance have been investigated (Brenta, 2011; Zhang et al., 2017; Gao et al., 2021), and merit additional AOP development efforts. The recently developed AOP ID 457 linking succinate dehydrogenase inhibition to increased insulin resistance through reduction in circulating thyroxine could be used as a basis to expand on this. A second priority is related to MIEs that are currently missing in the AOP network, such as thyroid hormone transporter inhibition (Noyes et al., 2019; Di Cosmo et al., 2022). The addition of such MIEs will improve the description of paths leading to downstream AOs/diseases. A third priority focuses on the molecular mechanism of crosstalk between estrogen and AhR signaling pathways leading to immunosuppression (Groestlinger et al., 2022), and linking to the work in Case study 1. Overall, the linkage between endocrine disruption and impaired immune function is an important area where AOPs are currently missing. A fourth priority focuses on expanding the available AOPs linking THSD to DNT. So far, focus in this area has been on impaired function of the hippocampus, and inclusion of impaired development of the hypothalamus leading to impaired motoric activity or social responses has been identified as a priority. For example, Harder et al. (2018) found that maternal thyroid hormone is required for parvalbumin neuron development in the anterior hypothalamic area. While such AOPs are not considered to lead to endocrine diseases *per se* and the existing AOPs leading to DNT (e.g., AOP IDs 42 and 54) have therefore not been included in the list of AOPs linking to diseases in Case study 2, they are addressed in detail in Case study 3. Across all AOP development efforts, attention will go to the comparison of pathways in mammalian and non-mammalian species including invertebrates to on the one hand advance the use of alternatives to animal testing such as fish embryos and invertebrate models for informing on human health, and on the other hand improve the use of mammalian data such as those of human *in vitro* systems for informing on environmental health.

#### 3.5.3 Case study 3: Neurotoxicity AOPs in the AOP-Wiki and their human health relevance

Neurotoxicity encompasses the study of adverse effects on the structure or function of the developing or adult (mature or aging) nervous system following acute or chronic exposure to chemical, biological, or physical agents (EPA, 1998; FDA, 2000). The distinction between developmental and adult neurotoxicity is fundamental due to the higher and/or different susceptibility of the highly plastic developing *versus* the poorly regenerative adult brain. Also, the role of signaling molecules guiding brain development differs as a function of the neurodevelopmental window. This distinction between developmental and adult neurotoxicity is also reflected in the AOPs, and the two case studies are discussed separately here.

##### 3.5.3.1 Developmental neurotoxicity (DNT)

In total, 12 AOPs were identified for DNT in the AOP-Wiki, as listed in Supplementary Table S7 (sheet 1 DNT AOPs). According to ICD-11, AOP IDs 12, 13, 17, 42, 54, 134, 152, 300 and 442 can be classified under ‘06 *Mental and Behavioural Disorders; neurocognitive disorders*’, which address impairment of learning and memory, and decreased cognitive function. Furthermore, AOP ID 12 “*Chronic binding of antagonist to N-methyl-D-aspartate receptors (NMDARs) during brain development leads to neurodegeneration with impairment in learning and memory in aging*” can also be classified under “*Diseases of the nervous system*” since early life disturbances may lead to adverse effects later in life. Although some cognitive disorders are covered by the above-mentioned AOPs in the AOP-Wiki, motor-function-related disorders (ICD-11 6A04 Developmental motor coordination disorder) represent one of the AOP-Wiki AO gaps. Such AOPs were previously hypothesized in the literature focusing, e.g., on the MDMA-induced decreased motor function in children (Barenys et al., 2020) and would merit further development and upload in the AOP-Wiki.

**FIGURE 8:**
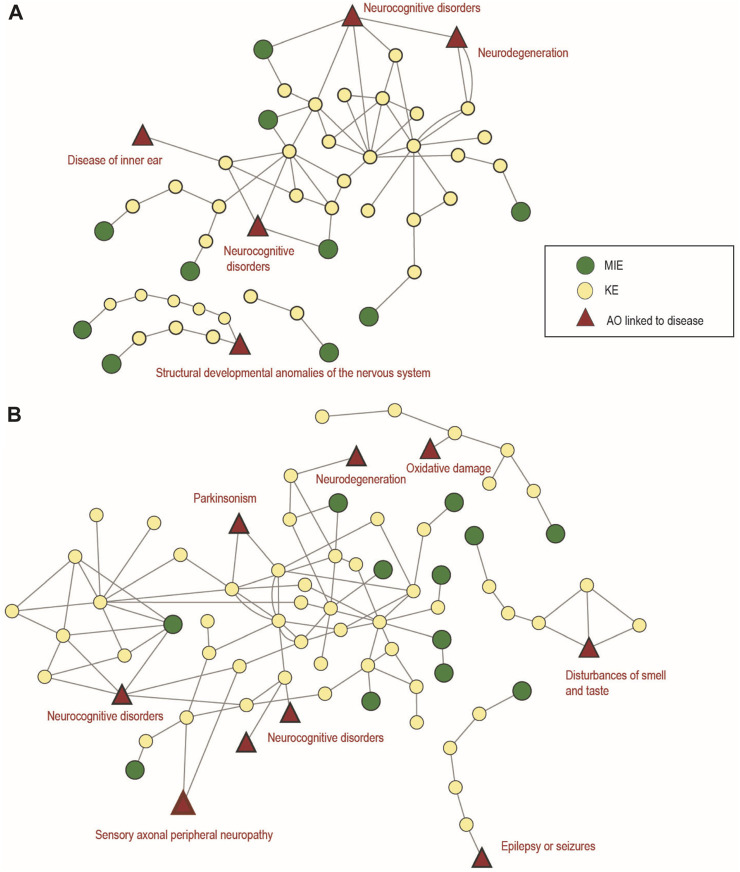
Adverse Outcome Pathway Networks (AOPN) for developmental and adult neurotoxicity. **(A)** AOPN for 12 AOPs linked to developmental neurotoxicity, identified in the AOP-Wiki. **(B)** AOPN for 14 AOPs linked to adult neurotoxicity, identified in AOP-Wiki. In both (A) and (B) Figures, the relevant ICD-11 disease category is annotated in red. Non-human relevant and empty AOPs have been excluded.

After exclusion of duplicate MIEs (Supplementary Table S7 sheet 2 DNT MIEs) and KEs (Supplementary Table S7 sheet 3 DNT KEs), a total of 10 MIEs and 41 kEs have been identified in the DNT context. Several of these KEs also visualized in Figure 8 constitute well-described factors influencing the onset and progression of neurological and neurodevelopmental disorder (NDD) pathologies. A large group of KEs (KE IDs 277, 281, 280, 425, 958, 959, 960, 1656, and 279) related to thyroid disruption and presents a direct link with chemically-induced congenital hypothyroidism (ICD-11 5A00) (Korevaar et al., 2016; Sun et al., 2022). KE IDs 1502 and 1503 characterizing defective histone acetylation were established as key regulators of processes leading to neural tube defect cases such as spina bifida (ICD-11 LA02) and anencephaly (ICD-11 LA00) (Tamkeen et al., 2021; Takla et al., 2023). Most importantly, KE ID 381 represents the brain-derived neurotrophic factor (BDNF), a key molecule necessary for healthy brain development and notably its learning and memory function. The dysregulation of BDNF was linked with numerous NDDs such as Autism Spectrum Disorder (ASD, ICD-11 6A02) (Liu et al., 2021), dyslexia (ICD-11 6A03) (Abdelraouf et al., 2023) and intellectual disability (ICD-11 6A00) (Esnafoglu and Adıgüzel, 2021), but not with Attention Deficit Hyperactivity Disorder (ADHD, ICD-11 6A05) (Mei et al., 2022; de Lucca et al., 2023). ADHD was, on the other hand, associated with impaired synaptogenesis (KE ID 385) (Dark et al., 2018; Halperin et al., 2021). At last, the downstream KE 386 of the decreased neural network function is a hallmark of many cognitive disorders including fetal alcohol syndrome, ASD or intellectual disability amongst others (Uhlhaas and Singer, 2006; Frega et al., 2020; Adams et al., 2022; McCready et al., 2022). Mapping neurodevelopmental KE from AOPs to human NDD will increase certainty for their regulatory application. For this purpose, human-relevant *in vitro* assays like the ones that are present in (Crofton and Mundy, 2021) or currently developed for the DNT IVB are valuable tools for filling the large data gap on DNT modes-of-action for large numbers of so far for DNT untested chemicals (Sachana et al., 2021).

Despite the fact that diseases of the nervous system are not underrepresented in the AOP-Wiki (r = 0.78; *p* = 0.435; Figure 5), it is well established that there are challenges in developing AOPs for neurotoxicity following neurodevelopmental exposure (Bal-Price et al., 2017), e.g., the lack of understanding of the MIEs that are causally responsible for triggering downstream KEs resulting in cognitive defects. One reason for this poor knowledge specifically when it comes to MIEs relevant for human NDD is the inability to study such events in humans and the poor representation of the spectrum of features of NDD in rodents (Paparella et al., 2020). Moreover, there are considerable comorbidities among NDDs (Surén et al., 2012) as well as several phenotypic similarities such as problems with language, cognitive function, social interaction, and attention across different NDD. Also here human-relevant, multicellular *in vitro* assays provide an exceptional opportunity for gaining information on the broad spectrum of MIEs that have the ability to trigger NDD in humans (Hogberg and Smirnova, 2022; Klose et al., 2022).

The onset of these disorders stems from alterations in the precisely orchestrated processes at different stages of human brain development. Despite its complexity, key neurodevelopmental processes that are vital for normal brain development have been identified and a majority of them are assessed in the DNT *in vitro* battery (IVB) (Crofton and Mundy, 2021). Although well established as KEs in brain development and DNT processes as well, several of these key neurodevelopmental processes (e.g., migration, neurite outgrowth, neuronal maturation, glia cell and neuronal differentiation) are currently not described in the AOP-Wiki and therefore illustrates major gaps that need filling. The following KEs that are so far not represented in the AOP-Wiki will enable AOP mapping of gene-environmentally induced NDDs such as microcephaly (ICD-11 classified LD20.2) caused by decreased NPC-proliferation KE (de Groot et al., 2005), ASD (ICD-11 6A02) linked to synaptic pruning KE (Pagani et al., 2021), as well as disorders linked to deficient autophagy KE (Deng et al., 2021). Mapping molecular and/or cellular features of human NDD to AOPs will strongly increase confidence in their regulatory application.

Although dysfunction of glial cells contributes to the pathogenesis of NDDs, KEs linked to their impaired development are mostly lacking from the AOP-Wiki. Thus, a KE of deficient astrocyte development, that is currently deficient in the AOP-Wiki, could be linked to intellectual disabilities (ICD-11 6A00) (Cresto et al., 2019), KE of radial glia development leading to simplified gyral appearance and reduced cortical surface area could be associated with Down syndrome (ICD-11 LD40.0) (Baburamani et al., 2020), and KE of impaired oligodendrocyte development with subsequent hypomyelination KE could be connected to perinatal white matter injury (ICD-11 KB02) (Murphy et al., 2007; Volpe et al., 2011; Motavaf and Piao, 2021). Furthermore, endocrine disruption-related KEs and MIEs absent from the DNT-related AOPs in the AOP-Wiki (e.g., binding to estrogen, androgen, liver x, and retinoic acid receptors) represent another major data gap preventing the linkage of cognitive disorders observed in children after prenatal exposure to endocrine disruptors (Lupu et al., 2020; Cediel-Ulloa et al., 2022) or fetal alcohol spectrum disorder (ICD-11 6A0Y) (Petrelli et al., 2019). By filling these data gaps in the future, we aim at providing a solid basis of scientific confidence to regulatory agencies that allows application of NAMs for DNT in a regulatory context. Considering that currently testing chemicals for DNT is not mandatory, an AOP-based NAM approach to broad-based substance testing is being pursued to protect the highly sensitive brains of our future generations.

##### 3.5.3.2 Adult neurotoxicity (ANT)

The development of an AOPN from all currently available linear ANT AOPs in the AOP-Wiki requires a thorough analysis and expert knowledge to clean up and harmonize the definition of KEs and even AOs, as often different terminologies are used for the same (or very similar) KE or AO description, which can be merged. In this case study, the only curation performed was to consider only fully or partially developed AOPs as part of the network, excluding empty AOPs (Figure 8B). As a result, 14 AOPs (11 partially drafted and 3 completed) referred to ANT in the AOP-Wiki database.

Five AOPs are included in the OECD work plan (AOP IDs 3, 10, 48, 394 and 475) and 3 of these are endorsed (AOP IDs 3, 10 and 48). According to the general category identified in the ICD -11, 4 AOPs (AOP IDs 48, 405, 475, 483) can be classified under ‘*Mental and Behavioural Disorders*’ with particular reference to ‘Neurocognitive Disorders (6D71)', which address learning and memory impairments and refer to more cognitive domains that represent a decline from the individual’s previous level of functioning (Figure 8B). Eight AOPs can be classified in ‘*Diseases of the nervous system*’ with reference to Parkinson’s disease (8A00) (AOP IDs 3 and 464), Alzheimer’s disease (8A20) (AOP ID 429), epilepsy or seizures (AOP IDs 10 and 281), disorders of nerve roots, plexus or peripheral nerves, covering peripheral neuropathy of sensory neurons (AOP IDs 279 and 450) or disorders of olfactory nerves (AOP ID 394) (Figure 8B). Three AOPs (AOP IDs 26, 260 and 281) do not refer to specific disease of the nervous system since addressing general neurotoxic effects such as oxidative damage or neurodegeneration (necrosis or apoptosis) (Figure 8B).

Motor deficit disorders, some of which are coded in ICD-11, such as ataxia (8A03), dystonia (8A02), myoclonus (8A06), choreoathetosis (8A01) and weakness, flaccid/spastic paralysis in addition to delayed neuropathy, psychosis and emotional disturbance, visual impairment (9D90) and hearing loss are among the adult nervous system dysfunctions resulting from exposure to toxic substances (Klaassen, 2018) that are not currently developed in the AOP-Wiki database.

A systematic review conducted in 2017, covering 27 years of literature, identified a set of key endpoint categories induced by human neurotoxicants and associated with ANT (Masjosthusmann et al., 2018). These categories relate to neurotransmission (cholinergic, GABAergic, glycinergic, glutamatergic, adrenergic, serotonergic, dopaminergic, neurotransmission in general), ion channels/receptors (sodium channels, potassium channels, calcium channels, chloride channels, other receptors), cellular endpoints (mitochondrial dysfunction/oxidative stress/apoptosis, redox cycling, altered calcium signaling, cytoskeletal changes, neuroinflammation, axonopathies, myelin toxicity, delayed neuropathy, enzyme inhibition) (Masjosthusmann et al., 2018). MIEs and KEs associated with AOPs that are fully or partially described in the AOP-Wiki database were organized according to these and related categories (Supplementary Table S7 sheet 4 ‘ANT endpoint categories ‘and sheet 5 ‘Additional ANT endpoints’). It is clear from these tables that domains such as ‘ion channels’ and part of the neurotransmitter system targeted by neurotoxicants are completely neglected (Supplementary Table S7 sheet 6 ANT endpoints not covered), although some of these, e.g., increase/inhibition of dopaminergic neurotransmission, are described in AOPs linked to obesity (ID 72) epithelial tumors (ID 170), malignant neoplasms of female genital organs (ID 112) and diseases of the genital system (ID 73) (Supplementary Table S2). According to the OECD AOPs Developer’s Handbook (OECD, 2018), KEs should be described as single isolated measurable events in order to be modular and to be used in other AOPs. These KEs can thus be reused to develop AOPs that address ANT.

Another aspect that emerges is the focus of AOPs on neurons. It is now well accepted that glial cells are key players in the control of nervous system homeostasis and that dysfunction of this cell group plays a role in neurological disorders (Jäkel and Dimou, 2017; von Bernhardi et al., 2016). With the sole exception of neuroinflammation, AOPs targeting glial cell toxicity (i.e., astrocytes, microglia, oligodendrocytes/Schwann cells) in the context of ANT are lacking.

From a regulatory point of view, a major challenge for ANT is the long-term health effects arising from repeated low-level exposure. For example, meta-analyses suggest an association between pesticide exposure and neurodegenerative diseases such as Parkinson’s disease, Alzheimer’s disease and amyotrophic lateral sclerosis (Malek et al., 2012; EFSA, 2013; Ntzani et al., 2013; Yan et al., 2016; Shrestha et al., 2020; Andrew et al., 2021). Thus, although epidemiological studies do not prove causality, they raise concerns and questions about the adequacy of *in vivo* regulatory studies to provide information on complex human health outcomes. Parkinson’s disease is already addressed by AOP IDs 3 (endorsed) and 464. In this context, to facilitate the functional understanding of complex biological systems, the curation of the ANT network and the development of AOPs addressing key steps leading to Alzheimer’s disease, with particular attention to the data gaps described, have been identified as priorities in PARC.

#### 3.5.4 Adverse outcome pathway network of the three prioritized endpoints of PARC

**FIGURE 9:**
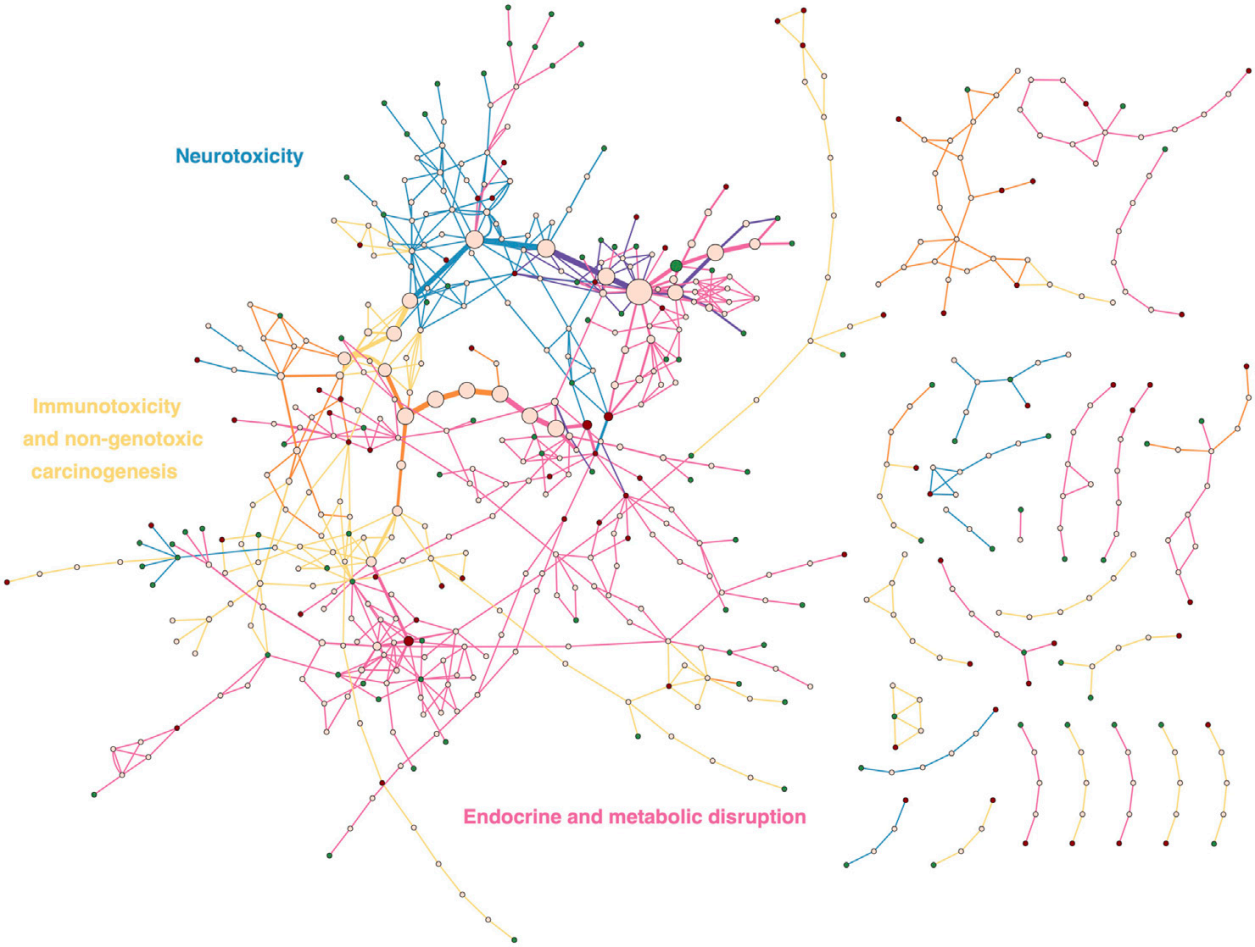
AOP network consisting of all AOPs inventori ed in the areas of the three prioritized endpoints of PARC, corresponding to the three case studies. KERs related to immunotoxicity and non-genotoxic carcinogenesis (Case study 1) are shown in yellow, KERs related to endocrine and metabolic disruption (Case study 2) are shown in pink and KERs related to neurotoxicity (case study 3) are shown in blue. KERs that are shared between immunotoxicity and non-genotoxic carcinogenesis and endocrine and metabolic disruption are shown in orange. KERs that are shared between neurotoxicity and endocrine and metabolic disruption are shown in purple. MIEs are shown in green and AOs are shown in red. Node size shows directed stress and edge width shows directed betweenness on uncurated subnetworks, calculated using the Cytoscape plugin CentiScaPe.

The three case studies were developed in the context of three priorities that have been set in the PARC project for AOP development in the areas of immunotoxicity and non-genotoxic carcinogenesis, endocrine and metabolic disruption, and neurotoxicity. Figure 9 shows an AOP network combining all AOPs that have been identified as related to immunotoxicity and non-genotoxic carcinogenesis (yellow in Figure 9, related to Case study 1), endocrine and metabolic disruption (pink, related to Case study 2) and neurotoxicity (blue, related to Case study 3). Τhere is a particularly well-connected area in the network where both the directed stress of the KEs (shown as larger node size in Figure 9) and the directed betweenness of the KERs (shown as larger edge width) are higher. Stress and betweenness consider the number of shortest paths that must pass through the KE/KER in question (Scardoni et al., 2009; Villeneuve et al., 2018a). This area connects the AOPs available in the AOP-Wiki in the three PARC priority areas. Neurotoxicity is strongly connected to endocrine and metabolic disruption through reduced thyroxine levels leading to reduced levels of BDNF. Immunotoxicity and non-genotoxic carcinogenesis is strongly connected to endocrine and metabolic disruption through DNA damage linked to increased activation of NF-kB and estrogen receptor antagonism, eventually resulting in metastatic breast cancer (AOP ID 443). As is the case for the three case studies separately, this overarching AOP network analysis is limited to those AOPs that have been entered into the AOP-Wiki by the community and to the level of curation and overall quality that is currently available in the AOP-Wiki (See Conclusion section for more details on related challenges). As such, this analysis provides a state of the art of the available AOPs and especially of connections between these areas that have been described so far. It should be noted that the linkages between the three case studies that can be discerned at this point are not necessarily the most important linkages, from a biological and toxicological point of view, among these research areas. Additional linkages may be investigated in the future, such as a link between thyroid dysfunction and Alzheimer’s disease (Kim et al., 2022).

## 4 Discussion/conclusion

The AOP framework supports a better description of evidence-based pathways leading to diseases, and it provides robust knowledge on critical key events and relationships that can lead to the development of novel tests and relevant biomarkers. In this study, we provide an overview of the existing AOPs available in the AOP-Wiki, generalized by overrepresentation analysis, highlighting their significance in advancing our understanding of toxicological mechanisms and informing risk assessment practices (Mortensen et al., 2022). We have presented the general characteristics of over- and under-represented AOPs in the AOP-Wiki and established possible connections to existing diseases (defined by ICD-11 categories) through a combination of computational tools and expert curation. In addition, we supplemented details about three case studies, prioritized within the PARC project, to shed light on the findings from this computational exercise.

One of the main outcomes of this study is that there is a large difference between the number of AOPs mapped to certain diseases as compared to other ones. There may be trivial reasons for that such as the focus of the groups who first developed AOPs. Yet, some critical diseases such as cardiovascular, blood, skin diseases or gut diseases appear to be underrepresented in the AOP-Wiki despite their considerable contribution to mortality and morbidity. This does not mean that the effort concerning well represented diseases such as genitourinary diseases or neoplasms should decrease as we are still far from a full description of pathways in these diseases, but rather that more effort should be devoted to underrepresented or neglected diseases. A stronger interaction between scientists involved in these diseases and those involved in AOP development should be helpful.

Another reason for explaining the disparity in AOPs may stem from the assumption we made regarding the theoretical distribution of the disease classes. Indeed, we assumed that the distribution would be uniform, i.e., that all categories would be strictly equal in terms of the AOPs that make them up. However, as AOPs are a framework strongly associated with toxicology and the environment surrounding us, since each MIE arises from a stressful exposure, it may be conceivable that the unknown set of all existing stressors disrupts only a limited number of biological functions, unevenly, as a subset of all existing pathologies. In this case, the distribution of ICD-11 classes should not be uniform, rather an asymmetrical distribution, to reflect the situation more accurately. For instance, this hypothesis would not attribute such a high negative weight to the sleep wake disorders class, which is currently significantly under-represented. Accordingly, it could have a more moderate weight because this physiological function is less disturbed by stressors. On the other hand, it might be expected that genitourinary diseases, neoplasms, and developmental diseases are genuinely over-represented since stressors lead to these pathologies more than any others. Moreover, the unequal distribution of AOPs with respect to human diseases may also be due to the fact that some biological spaces are over-represented, such as fundamental function which appear to be common and involved in various processes. A bias in coverage of diseases, originating from a database used, its versions or granularity could also be expected. Here we used the latest WHO database, that is a world-wide recognized, curated database of human diseases but other existing databases with human diseases definition could potentially depict slightly different patterns, reflecting a historical aspect of naming and characterization of human diseases.

Further, our study revealed that the genes that are strongly connected with the disease classes differ from those that are most frequently found in the AOPs. There are several explanations for this observation, but it does suggest that some important disease pathways in current AOPs are missing and that efforts should be devoted to developing additional AOPs involving genes that are highly connected to diseases. It can be further explained by the fact that the structure of ICD-11 and disease categories consider different ontology and semantics compared to AOP-Wiki (ICD-11 is more related to genetic phenotypes and clinical data and AOP-Wiki more focused on adverse outcomes).

Relative to the AOP framework itself, our analysis illustrates a number of aspects and challenges that are important in the context of the currently ongoing effort to increase the FAIRness (Findability, Accessibility, Interoperability, and Reusability) of the AOP-Wiki (Wittwehr et al., 2023) and of toxicological data in general as emphasized by the recently established European intergovernmental ELIXIR toxicology community (Martens et al., 2021). For example, for efforts such as the present analysis where AOPs are collected through the AOP-Wiki and assembled into AOP networks to be used for meta-analyses, a strong need for AOP (network) curation arises, such as the merging of synonymous KEs to reduce redundancy, the grouping of related but not necessarily identical KEs, and the unambiguous description and identification of KERs. In this context the use of ontological annotations in the AOP-Wiki is becoming increasingly important (Ives et al., 2017; Wittwehr et al., 2023) and is envisioned as one of the cornerstones of the data model that is being developed for the AOP-Wiki version 3.0, which aims to address many of these challenges. This will increase the interoperability with other databases and platforms and facilitate analyses such as the present one.

Also, the development of large AOP networks results in the emergence of new linear AOPs by re-using building blocks from user defined AOPs (Pollesch et al., 2019). An important challenge lies in inventorying and assessing the quality of such emergent AOPs. New tools to address this challenge are underway, which will allow the prioritization of emergent AOPs for further manual assessment and consideration. Another aspect that becomes especially important when assembling AOPs into AOP networks is how to deal with domain of applicability (DOA) descriptions (currently limited to taxonomic group, life stage and sex, but potentially also including other components such as tissue and organ type) across interconnecting AOPs. The DOA has often not been thoroughly investigated for many users defined AOPs, and determining the DOA becomes even more complex in the case of emergent AOPs. For example, an AOP may be established in animals but its relevance to humans may not have been explored. For that reason, not only documenting evidence of conservation across species, but also including evidence that a certain KE/KER is not conserved in a particular taxon may become an important tool for analyzing large AOP networks. A deeper analysis of the KER would also be interesting for further exploration in terms of relevance to human diseases, and to gain a better understanding of the biological space.

Finally, the AOP concept serves as a powerful tool for the systematic classification of knowledge, offering immense potential within the regulatory landscape. By providing a structured framework to understand complex pathways leading to AOs that we can connect to diseases, AOPs will enable more informed decision-making and risk assessment. Furthermore, the emphasis on identifying gaps in our understanding paves the way for the targeted development of assays that address these areas. This dynamic approach encourages innovative thinking, allowing us to explore uncharted territories in toxicology. As we harness the full scope of AOPs, we not only enhance our comprehension of biological interactions but also shape a future where predictive toxicology is both accurate and comprehensive.

## Data Availability

The datasets presented in this study can be found in online repositories. The names of the repository/repositories and accession number(s) can be found in the article/Supplementary Material.
