# Supplementary material for: Comprehensive mapping of the AOP-Wiki database: identifying biological and disease gaps
Source: Front Toxicol. 2024 Mar 8;6:1285768. doi: 10.3389/ftox.2024.1285768 (PMC10958381; doi:10.3389/ftox.2024.1285768)
Supplement: Supplementary file 1 [file DataSheet1.docx]

Supplementary Material

Comprehensive mapping of the AOP-Wiki database: identifying biological and disease gaps.

Thomas Jaylet, Thibaut Coustillet, Nicola M. Smith, Barbara Viviani, Birgitte Lindeman, Lucia Vergauwen, Oddvar Myhre, Nurettin Yarar, Johanna M. Gostner, Pablo Monfort-Lanzas, Florence Jornod, Henrik Holbech, Xavier Coumoul, Dimosthenis A. Sarigiannis, Philipp Antczak, Anna Bal-Price, Ellen Fritsche, Eliska Kuchovska, Antonios K. Stratidakis, Robert Barouki, Min Ji Kim, Olivier Taboureau, Marcin W. Wojewodzic, Dries Knapen, Karine Audouze

# Supplementary Figures and Tables

## Supplementary Figures

**Supplementary Figure 1.** Representation of the most studied genes/proteins in the current AOPs present in the AOP-Wiki database (as of May 2023).

**Supplementary Figure 2.** Representation of the most relevant MFs after clustering based on semantic similarity and multidimensional scaling using ReviGO. The size of the circle is proportional to the size of each GO term, while the color represents the adjusted p-value (log10). The following settings were used: Default settings; Work With (Homo Sapiens); Resulting list (Tiny).

**Supplementary Figure 3.** Barplot of the top 25 most significant diseases from the CURATED DisGeNET database based on their Gene Ratio. The x-axis displays the gene ratio of each Disease, and the y-axis lists the descriptions of the respective disease name. The bars are filled with colors according to their adjusted p-values, with lighter shades indicating lower p-values and higher significance.

**Supplementary Figure 4.** Chord diagram illustrating the associations between diseases and genes. Diseases are represented on the bottom side of the diagram, while genes are displayed on the top side. The width of the chords represents the strength of the association between a disease and a gene, with wider chords indicating stronger associations. For clarity, only associations with more than 60% involvement are shown.

**Supplementary Figure 5.** Representation of the top 30 most studied Molecular Initiating Events (MIEs) in the AOP-Wiki database (as of May 2023).

**Supplementary Figure 6.** Representation of the top 30 most studied Key Events (KEs) in the AOP-Wiki database (as of May 2023).

**Supplementary Figure 7.** Representation of the top 30 most studied Adverse Outcomes (AOs) in the AOP-Wiki database (as of May 2023).

**Supplementary Figure 8.** Clustering of the 149 distinct Adverse Outcomes (AOs) in AOP-wiki after merging of synonyms (as of May 2023). The merged terms are stored in Table S2 sheet 6 *synonyms*.

**Supplementary Figure 9.** Distribution of the 17 AOPs classified as ‘*Endocrine, nutritional or metabolic diseases*’ by the IDC-11 classification systems.

## Supplementary Tables

**Supplementary Table 1.** Genes and proteins extracted from the biological events of AOP-Wiki (as of May 2023).

**Supplementary Table 2.** AOP-Wiki Adverse Outcomes Classification using the 11th International Classification Diseases system (ICD-11) provided by the World Health Organization (as of May 2023). Sheet 1: Classification of the 149 distinct AOs. Sheet 2: Distribution of ICD-11 categories for the 149 distinct AOs. Sheet 3: Classification of AOPs. Sheet 4: Number of AOPs in which AOs are found. Sheet 5: Number of AOs in each AOP. Sheet 6: Merged AOs that are actually synonymous. Sheet 7: Statistical test.

**Supplementary Table 3.** GO enrichment results using g:profiler for Biological Process (BP ; sheet 1), Molecular Function (MF; sheet 2) and Cellular Component (CP; sheet 3).

**Supplementary Table 4.** Disease Enrichment Analysis Results from DisGeNET Database.

**Supplementary Table 5.** Supplementary data related to Case study 1 (Immunotoxicity and Non-Genotoxic Carcinogenesis). Sheet 1: Common and specific events of AOP ID 39 (*Covalent Binding, Protein, leading to Increase, Allergic Respiratory Hypersensitivity Response*) and AOP ID 40 (*Covalent Protein binding leading to Skin Sensitisation*). Sheet 2: AOPs identified from the AOP-Wiki related to Non-genotoxic carcinogenesis. Of the 45 AOPs listed, 32 were complete enough to be visualised in the Adverse Outcome Pathway Network and have their relevant ICD-11 disease subcategory specified. Only AOPs leading to an Adverse Outcome related to cancer and applicable to mammals were included.

**Supplementary Table 6.** Supplementary data related to Case study 2 (Endocrine and Metabolic Disruption). Listing all AOPs leading to endocrine and metabolic diseases. Disease subcategories were used as labels in the AOPN visualization in Figure 7. Disease names between brackets have been added for clarity and ease of visualization.

**Supplementary Table 7.** Supplementary data related to Case study 3 (Developmental and Adult Neurotoxicity). Sheet 1: Developmental neurotoxicity AOPs identified in the AOP-Wiki. Sheet 2 : MIEs for neurodevelopmental AOPs identified in the AOP-Wiki. Sheet 3: KEs for neurodevelopmental AOPs identified in the AOP-Wiki. Sheet 4: ANT endpoint categories covered in the AOP-Wiki database. Sheet 5: Additional ANT endpoint categories covered in the AOP-Wiki. Sheet 6: ANT endpoint categories not covered in the AOP-Wiki.

**Supplementary Figure 1.** Representation of the most studied genes/proteins in the current AOPs present in the AOP-Wiki database (as of May 2023).

**Supplementary Figure 2.** Representation of the most relevant MFs after clustering based on semantic similarity and multidimensional scaling using ReviGO. The size of the circle is proportional to the size of each GO term, while the color represents the adjusted p-value (log10). The following settings were used: Default settings; Work With (Homo Sapiens); Resulting list (Tiny).

**Supplementary Figure 3.** Barplot of the top 25 most significant diseases from the CURATED DisGeNET database based on their Gene Ratio. The x-axis displays the gene ratio of each Disease, and the y-axis lists the descriptions of the respective disease name. The bars are filled with colors according to their adjusted p-values, with lighter shades indicating lower p-values and higher significance.

**Supplementary Figure 4.** Chord diagram illustrating the associations between diseases and genes. Diseases are represented on the bottom side of the diagram, while genes are displayed on the top side. The width of the chords represents the strength of the association between a disease and a gene, with wider chords indicating stronger associations. For clarity, only associations with more than 60% involvement are shown.

**Supplementary Figure 5.** Representation of the top 30 most studied Molecular Initiating Events (MIEs) in the AOP-Wiki database (as of May 2023).

**Supplementary Figure 6.** Representation of the top 30 most studied Key Events (KEs) in the AOP-Wiki database (as of May 2023).

**Supplementary Figure 7.** Representation of the top 30 most studied Adverse Outcomes (AOs) in the AOP-Wiki database (as of May 2023).

**Supplementary Figure 8.** Clustering of the 149 distinct Adverse Outcomes (AOs) in AOP-wiki aftermerging of synonyms (as of May 2023). The merged terms are stored in Table S2 sheet 6 *synonyms*.

**Supplementary Figure 9.** Distribution of the 17 AOPs classified as ‘*Endocrine, nutritional or metabolic diseases*’ by the IDC-11 classification systems.

**Supplementary Table 1.** Genes and proteins extracted from the biological events of AOP-Wiki (as of May 2023).

[Tables S1](https://docs.google.com/spreadsheets/d/12p7__NzpOfckEgEDmp9yhegxkpcGOdBk/edit?usp=drive_link&ouid=102626691237344149745&rtpof=true&sd=true)

**Supplementary Table 2.** AOP-Wiki Adverse Outcomes Classification using the 11th International Classification Diseases system (ICD-11) provided by the World Health Organization (as of May 2023). Sheet 1: Classification of the 149 distinct AOs. Sheet 2: Distribution of ICD-11 categories for the 149 distinct AOs. Sheet 3: Classification of AOPs. Sheet 4: Number of AOPs in which AOs are found. Sheet 5: Number of AOs in each AOP. Sheet 6: Merged AOs that are actually synonymous. Sheet 7: Statistical test.

[Tables S2](https://docs.google.com/spreadsheets/d/1MayezQR_cbj3JEFOoCrGwomBKaWq3Mek/edit?usp=drive_link&ouid=102626691237344149745&rtpof=true&sd=true)

**Supplementary Table 3.** GO enrichment results using g:profiler for Biological Process (BP ; sheet 1), Molecular Function (MF; sheet 2) and Cellular Component (CP; sheet 3).

[Tables S3](https://docs.google.com/spreadsheets/d/1rliZRJeDkAzXWfNzGM8tOBIBzFN9VQM_/edit?usp=drive_link&ouid=102626691237344149745&rtpof=true&sd=true)

**Supplementary Table 4.** Disease Enrichment Analysis Results from DisGeNET Database.

[Tables S4](https://docs.google.com/spreadsheets/d/1_DIOG9WvZj8OSRHtjs2eLDiNmRG0Xcjv/edit?usp=drive_link&ouid=102626691237344149745&rtpof=true&sd=true)

**Supplementary Table 5.** Supplementary data related to Case study 1 (Immunotoxicity and Non-Genotoxic Carcinogenesis). Sheet 1: Common and specific events of AOP ID 39 (*Covalent Binding, Protein, leading to Increase, Allergic Respiratory Hypersensitivity Response*) and AOP ID 40 (*Covalent Protein binding leading to Skin Sensitisation*). Sheet 2: AOPs identified from the AOP-Wiki related to Non-genotoxic carcinogenesis. Of the 45 AOPs listed, 32 were complete enough to be visualised in the Adverse Outcome Pathway Network and have their relevant ICD-11 disease subcategory specified. Only AOPs leading to an Adverse Outcome related to cancer and applicable to mammals were included.

[Tables S5](https://docs.google.com/spreadsheets/d/14rfIXYNGozWzjrO2k-BtY5FjOg86JqSV/edit?usp=drive_link&ouid=102626691237344149745&rtpof=true&sd=true)

**Supplementary Table 6.** Supplementary data related to Case study 2 (Endocrine and Metabolic Disruption). Listing all AOPs leading to endocrine and metabolic diseases. Disease subcategories were used as labels in the AOPN visualization in Figure 7. Disease names between brackets have been added for clarity and ease of visualization.

[Tables S6](https://docs.google.com/spreadsheets/d/1737FbUPjKU3I0BoSVNYo8hWZTv7j476q/edit?usp=drive_link&ouid=102626691237344149745&rtpof=true&sd=true)

**Supplementary Table 7.** Supplementary data related to Case study 3 (Developmental and Adult Neurotoxicity). Sheet 1: Developmental neurotoxicity AOPs identified in the AOP-Wiki. Sheet 2 : MIEs for neurodevelopmental AOPs identified in the AOP-Wiki. Sheet 3: KEs for neurodevelopmental AOPs identified in the AOP-Wiki. Sheet 4: ANT endpoint categories covered in the AOP-Wiki database. Sheet 5: Additional ANT endpoint categories covered in the AOP-Wiki. Sheet 6: ANT endpoint categories not covered in the AOP-Wiki.

[Tables S7](https://docs.google.com/spreadsheets/d/11U4zfckY2SSMtq0N9-LEiJJasJZEjXQA/edit?usp=drive_link&ouid=102626691237344149745&rtpof=true&sd=true)
